# Validating a Thoroughbred Racehorse Welfare Index through Horse Behaviour and Trainers’ Reports of Welfare Issues in Their Horses

**DOI:** 10.3390/ani13020282

**Published:** 2023-01-13

**Authors:** Alison Glen Mactaggart, Clive Julian Christie Phillips

**Affiliations:** 1 Centre for Animal Welfare and Ethics, University of Queensland, Gatton, QLD 4343, Australia; 2 Institute of Veterinary Medicine and Animal Sciences, Estonian University of Life Sciences, Kreutzwaldi 46, 51006 Tartu, Estonia; 3 Curtin University Sustainable Policy (CUSP) Institute, Kent St., Bentley, WA 6102, Australia

**Keywords:** animal welfare, behaviour, horse racing, Thoroughbred racehorse, welfare index

## Abstract

**Simple Summary:**

Currently, no comprehensive horse welfare assessment system exists for Thoroughbred racehorse establishments in Australia, or anywhere in the world. We investigated the reliability of a welfare index that we developed from expert reports on major welfare issues. First, we observed the behaviour of racehorses in a range of training stables and found relationships to the index scores. Second, we surveyed horse trainers to understand how well they provided for the various welfare issues covered in the index. We conclude that the index could be used, with further validation, to evaluate and compare the welfare standards of training stables for their racehorses.

**Abstract:**

We validated a Thoroughbred racehorse welfare index, developed initially from expert opinion, by relating it to horse behaviour recorded in a range of training stables and surveying trainers to investigate the environment and management systems for Thoroughbred racehorses. Relationships between the index scores and horse behaviour were observed. Then, an Australia-wide survey of racing industry stakeholders was conducted to identify which parameters are important for welfare in a training stable. Trainers performed well on horsemanship and health/disease aspects. Provisions for ventilation, transportation and nutrition were also scored at high levels. However, provisions for weaning, wastage, assisting horses in coping with heat stress, stabling and the education of racehorses were not as well covered, indicating a need for improvement in these welfare issues. We concluded that our Thoroughbred Racehorse welfare index is able to discriminate between trainers offering differing levels of welfare for the major issues in racehorse training stables.

## 1. Introduction

The racing industry is continually challenged to improve Thoroughbred racehorse (TBR) conditions both in training and race procedures, particularly those identified as welfare issues. The training of TBRs is often based on management and husbandry methods that have been determined over many years to be convenient to humans but not necessarily scientifically evaluated. The deficiencies in the Thoroughbred racing industry are sometimes due to a lack of facilities, especially as old racecourses are increasingly surrounded by suburbia and lack adequate training and housing facilities for the TBR. Racetrack design (track shape, size and surface); appropriate housing (allowing adequate space and physical and visual contact for the TBRs); training methods; performance-related clinical problems; and transportation, including an adequate and safe area for horse transports on racecourses [1,2], are all welfare issues which need to be addressed. 

Thoroughbred racing is controlled in Australia by the Australian Racing Board (ARB), which was established in June 1998 [3], now Racing Australia Ltd (RAL)(Flemington, Victoria, Australia).. Though Thoroughbred horse racing in Australia is state-structured, there is overarching governance at the national level through RAL. The RAL, under its constitution, can make, change and administer the Australian Rules of Racing and “do all things whatsoever that the Board considers to be conducive to developing, encouraging, promoting or managing the Australian thoroughbred racing industry” [3]. This overarching governance at the national level, through the RAL, compliments local government legislation, enforcing codes of practice [4] where conformity to minimum standards and guidelines is required. However, minimum standards are not always able to address all of the major factors affecting the welfare of a stabled TBR, for example, the height of the stable must be in relation to the size of the TBR. In relation to this, Sainsbury [5] suggests stable sizes “for most horses of 4 m × 4 m” and for ponies of a smaller size (3.3 m × 3.3 m). The roof height recommended for barn-type stabling is “3 m at the eaves and rising to the ridge of about 8 m” [5].

The Australian Thoroughbred racing industry’s original concept of entertainment and recreation [3] has changed to become that of an industry with economic importance and, on a per capita basis, is currently the biggest racing industry in the world. This industry is dependent on the well-being of Thoroughbred racehorses. No uniform ranking or weighting system of welfare issues that are important for TBRs exists. We previously determined 14 key welfare issues for the TBR and ranked them based on the opinions of experts and a range of stakeholders. Other studies on equine welfare have been conducted [6,7,8,9,10], but none are specific to the management and welfare of the TBR, and many studies obtained data by using invasive methods that cannot be applied in practice.

As a result, we undertook to validate an index purporting to measure the welfare of TBRs using observations of TBR behaviour in a range of training stables and a survey of racehorse trainers relating to their provisions for the welfare of their horses. In conjunction with this, the time budgets of racehorses that we determined in a variety of stables were used to investigate how they related to the conditions in which they were kept and the horses’ welfare. 

## 2. Materials and Methods

### 2.1. Study 1—Horse Behaviour in Training Stables and Its Relation to TBR Welfare Index Scores

All observational procedures were approved by the University of Queensland Animal Ethics Committee, approval number 2011000067.

Thirty TBR racing trainers in Southeast Queensland were selected as having at least six racehorses in training from a list of all registered trainers in this region [11]. They were contacted with a request for permission to carry out behavioural studies at their training stables. Twenty-eight positive responses were received, of which two training stables were rejected, one having less than six horses in training, and the other due to timing difficulties, leaving twenty-six. Of these, 13 were chosen for behaviour data collection on the basis of having diverse stable designs and sizes, with and without the attachment of yards (Appendix A). A total of 156 TBRs were recorded, housed in 5 training stables in Brisbane and 8 in the Toowoomba and Westbrook areas in southeast Queensland, Australia. Mean TBR age was 3.5 years, SEM 0.134, median 3 years, range 1–9. There were 46 females (all entire) and 87 males (3 entire).

#### 2.1.1. Management of TBRs in the Surveyed Training Stables

All of the training stables had similar routines, with the day starting at about 0400 h when the TBRs were taken to the track for exercise. While the horses were out of their boxes, the stables were cleaned and fresh bedding was added. In most cases, the horses were stabled at the racecourse or had access to a private training facility; thus, there was no need for transportation. Those TBRs who were stabled away from the track were transported either by transport vehicle or a trailer float to an exercise facility. The racehorses were warmed up before commencing work, either by spending some minutes on a walking machine or by walking in an exercise area other than the racetrack. The racehorses were all worked at a fast pace on Tuesday and Thursday mornings. Some Tuesdays were set aside for practice starts or “jump outs”, which usually consist of three or four racehorses jumping out of the barrier (starting gate) and galloping for approximately 400–800 m. “Jump outs” form part of the early education of a racehorse, enabling the jockey and trainer to assess the level of race preparedness of the racehorse. Short race (barrier or starting gate) trials took place on Tuesdays, when “jump outs” were not being held, involving five to eight horses with a pace that is faster than for jump outs and jockeys wearing race colours, thus accustoming the horse to race day procedures. Barrier (starting gate) trials were used to assess the fitness of the TBR and the most suitable race distance for a particular racehorse.

After morning exercise, the TBRs were hosed down and attached to a rotary walking machine to cool and dry off for approximately 15 min before returning to their stables for feeding. The rest of the morning was spent resting in the stable until the afternoon, when they were either taken out to graze while held on a lead line or were provided with more exercise on the walking machine. Cleaning was repeated while the stables were empty. Upon completion of the stable cleaning process, the TBRs were returned to their stables for the evening meal, which consisted of concentrates and hay, sometimes with hay being offered again later in the evening. 

The training stable complex consisted of three distinct stabling categories commonly used for this purpose [12,13]:

1. Barn with central corridor and stables facing inward on both sides of the corridor.
2. Side-by-side stables opening outwards onto an open grassed area.
3. Yards with a roofed area, enclosed on one or more sides.

The smallest of the 13 stables was 3.3 × 3.7 m by 3 m high, and the largest was 5 × 5 m by 6.4 m high; mean size was 4.76 long by 3.96 m wide and 3.88 m high. Four training stables did not have yards attached to the stable or the use of a yard. Six of the nine training stables had yards attached to the stables, but one did not allow free use of the yards. Where possible, the TBRs were rotated between stable and yard, thus ensuring all horses had some hours each day out of their stables. In the case of the four establishments without yards, these TBRs were confined to their respective stables for 24 h per day, except for the time spent exercising on the track or on the walker.

#### 2.1.2. Behaviour Recording 

A continuous recording method was used for all occurrences of behaviour [14]. Each TBR was identified by their colour, sex and age at the commencement of observation. The age of each TBR was obtained from the year of birth brand. An ethogram was developed based on descriptions of the behaviour of equines in both stables [10,15,16] and free-range conditions [17,18]. Primary behaviour categories were determined as social, locomotion, oral, abnormal, repetitive behaviour, response to external stimuli and other [14]. This basic ethogram was extended to include an additional category, maintenance, which included feeding and drinking, elimination, lying, resting, sleeping, standing, rubbing and scratching (Table 1).

#### 2.1.3. Pilot Study

A pilot study was conducted with two observers continuously recording the behaviour of the same TBR. The two observers sat on a platform at a height of 900 mm, which allowed maximum visual exposure. Interobserver reliability was ensured by comparing behaviour assessments between the two observers, with modification of definitions as necessary. Each observer had a stopwatch to continuously record all individual behaviours occurring within each 15 min period. Twelve horses were observed in four groups of three stables at the following times: 0700–0800, 1100–1200 and 1500–1600 h. These 12 horses were not used in the subsequent behaviour study.

#### 2.1.4. Observation Method

Observations were conducted by the same two observers for the observational period, which ran from 24 April to 18 May. During this time, the minimum temperature ranged from 5.6 °C to a maximum of 24.4 °C. Of the 156 Thoroughbred racehorses for which recordings commenced, only 133 were used in the final analysis, as 23 horses were removed during the study to fulfil racing commitments.

The Thoroughbred racehorses were observed for three sessions each of 30 min in four groups of three horses by the two observers, from 0700–0800, 1100–1200 and 1500–1600 h. Viewing arrangements were the same as the pilot study, but at the completion of 15 min, the observers changed TBRs, minimizing observer bias and ensuring the continuous recording of the six horses under observation. Thus, each Thoroughbred racehorse in the study was observed for 90 min, and the total time for the observation of 133 Thoroughbred racehorses was 200 h.

#### 2.1.5. Ascribing a TBR Welfare Index (TBRWI) Score to Each Training Stable

Utility scores of high, middle or low for the TBR welfare issues examined in an expert opinion survey [40] were attributed to the 13 stables. These were then adjusted by issue importance values [40], determined based on the survey to provide each training stable with a percentage score. 

#### 2.1.6. Statistical Analysis

An initial analysis used stepwise regression, with forwards–backwards model fitting and alpha values of 0.15 to enter and remove variables. This was used to relate the TBRWI score attributed to each of the 13 stables to the mean value of the 26 behaviours recorded for all the focal horses in each stable. As one of the resulting correlated variables was clearly not linearly related to the TBRWI, a quadratic component was calculated for this variable. Pearson correlation coefficients were calculated for the 26 behaviours. A principal component analysis (PCA) was conducted on the log_10_ of behaviour values + 1 as a result of their nonnormal distribution. Age and gender effects were evaluated using analysis of variance of log_10_ transformed values of the behaviour + 1, with gender as a factor and age as a covariate. 

### 2.2. Part 2—Survey of Racehorse Training Stables’ Performance on the TBRWI

#### 2.2.1. The Survey and Its Distribution

The same fourteen issues and levels as used in the Thoroughbred racehorse training survey [40] were used in a survey of TBR trainers within Australia, who completed the survey for the horses they trained. Approval for the survey was obtained from the Human Ethics Committee of the University of Queensland (project number 2011000067). The survey (Appendix B) contained two sections. The first section in the survey consisted of 5 questions, requesting information on the respondent’s gender, age, education, length of involvement with TBRs and where they gained this experience. The second section of 14 questions sought information relevant to the TBRWI. It was created in vignette format [41,42] using survey software [43]. It initially asked the respondent how experienced their staff were, followed by a question regarding how well the health and disease problems of their TBRs were attended to. This was followed by questions concerned with the education of racehorses; the design and surface of racetracks; ventilation, both in the stable and in transporters; the amount of space their TBR had in their stable; and if there was a yard attached or not. Other questions dealt with weaning methods; the skill of transport drivers; nutrition and the availability of green forage; how the trainers coped with wastage of TBRs; whether their TBRs were exposed to extreme weather conditions; how often their TBRs were whipped in races; the environment in which the TBRs were kept; and, finally, how often tongue ties and or blinkers were used.

A link to the online survey was initially sent by email to 655 trainers within Australia, with addresses obtained from a previous survey. A letter accompanied the survey containing a brief explanation of the research project and instructions on how to complete the survey, as well as an approximate time for completion. Participants were advised of their right to withdraw at any time, who to contact if necessary and the opportunity to enter a draw for a prize. Most were returned unopened, so 405 new email addresses were sourced from websites and state racing directories. Only 71 were undeliverable. Finally, hard copies of the survey were sent to 250 trainers’ street addresses, obtained from racing journals, websites and state racing directories, together with a stamped addressed envelope. 

#### 2.2.2. Pilot Study

A pilot study was administered to three Thoroughbred racehorse trainers, and minor changes were made to the survey as a result.

#### 2.2.3. Statistical Analysis

Simple descriptive statistics were first determined. Relationships between issues were explored with a principal component analysis with a forwards–backwards fitting of components using the Minitab statistical package [44]. Likelihood ratio chi-squared analysis was used to determine the significance of gender differences in trainers’ responses. Some data for each question in the survey were too small for a statistical evaluation; when this occurred, they were combined with other levels in that question, but only when questions in each respective level were compatible with each other. 

The average importance scores and the average utility values for each of the 14 issue levels, as generated by the survey software [43], were used to calculate the values for the Thoroughbred Racehorse welfare index score. First, the average utility values were zero-centred. There were between two and four utility values for each importance score, which corresponds to the levels chosen for each Issue. The contribution of other levels was determined by scaling the utility values proportionately. By summing the utility values, a score of zero was obtained. The contribution of each utility value was weighted according to the importance score of each issue. The TBRWI was obtained using the following formula:

$$TBRWI=\sum_{i=1}^{14}\left(\frac{UV_{i}-Min_{i}}{Max_{i}-Min_{i}}\right)\frac{IS_{i}}{100}$$

where

*UV*_1_ = Individual Utility Value

*Min*_1_ = Minimum Utility Value

*Max*_1_ = Maximum Utility Value

*IS*_1_ = Issue Importance Score.

## 3. Results

### 3.1. Study 1—Horse Behaviour in Training Stables and Its Relation to TBR Welfare Index Scores

#### 3.1.1. Time Budgets and Interactions between Behaviours

Horses, on average, spent most of their time in just four behaviours, each occupying more than 5% of their time: feeding, standing, looking and standing inactive (Table 2). Coefficients of variation were relatively low; the highest were for rocking, chewing objects, licking, inactivity, resting, elimination and drinking. 

#### 3.1.2. Correlation Matrix and PCA

Drinking was negatively correlated with the locomotive stereotypies (Appendix C), particularly rocking (CC = −0.33, *p <* 0.001), but also head tossing (CC = −0.27, *p <* 0.001) and box walking (CC = −0.2, *p* = 0.02), and positively correlated with standing resting (CC = 0.24, *p =* 0.01). Similarly, feeding was highly negatively correlated with rocking (CC = −0.45, *p <* 0.001), windsucking (CC = −0.24, *p =* 0.01) and chewing objects (CC = −0.02, *p =* 0.02), but not correlated with resting (*p =* 0.61). Feeding was negatively correlated with walking (CC = −0.24, *p =* 0.01) and box walking (CC = −0.22, *p =* 0.01). Walking was positively correlated with weaving (CC = 0.25, *p <* 0.001). Elimination was positively correlated with aggression (CC = 0.24, *p <* 0.001) and sniffing (CC = 0.25, *p <* 0.001). As expected, sniffing was associated with pawing (CC = 0.19, *p* = 0.03), indicating the latter had an investigative function.

A group of correlated behaviours emerged from the matrix (Appendix C): rubbing, sniffing, playing, yawning, looking and aggression. Because of these apparent associations, the dataset was subjected to PCA.

**Figure 1:**
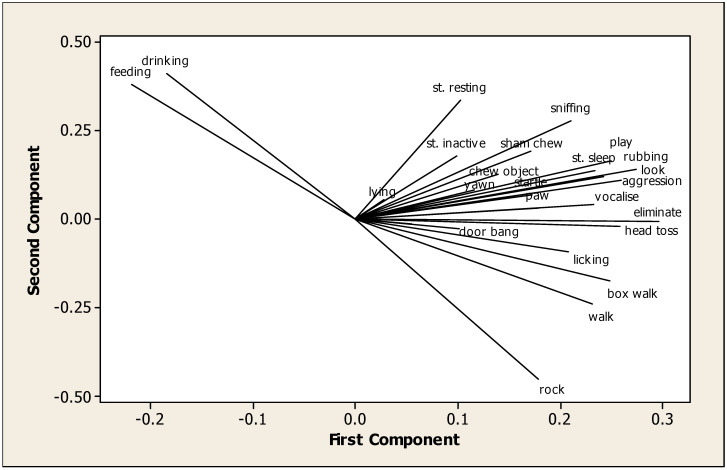
Behaviours in first and second components of principal component analysis. Resting = standing resting; standing = standing sleeping; inactivity = standing inactive.

The PCA produced ten components with eigenvalues over one, and we present the first two graphically (Figure 1). In this, feeding and drinking were closely related and antagonistic to weaving, walking and box walking. PC3 was concerned with oral stereotypies, windsucking/crib-biting, chewing objects, inactivity and standing (Table 3). In PC4, windsucking was negatively correlated with lying. In PC5, head tossing and sham chewing featured prominently, similarly to PC6 yawning and box walking; PC7 door banging, pawing and chewing objects; and PC 8 door banging and aggression.

#### 3.1.3. Gender and Age Differences 

Male horses spent much more time in elimination and rubbing compared with females (Table 4). No other behaviours were affected by gender. Windsucking (*p =* 0.003) and, to a lesser extent, box walking (*p =* 0.05) increased with the age of the horse (Appendix D).

#### 3.1.4. Correlations of Behaviours to the TBRWI

**Figure 2:**
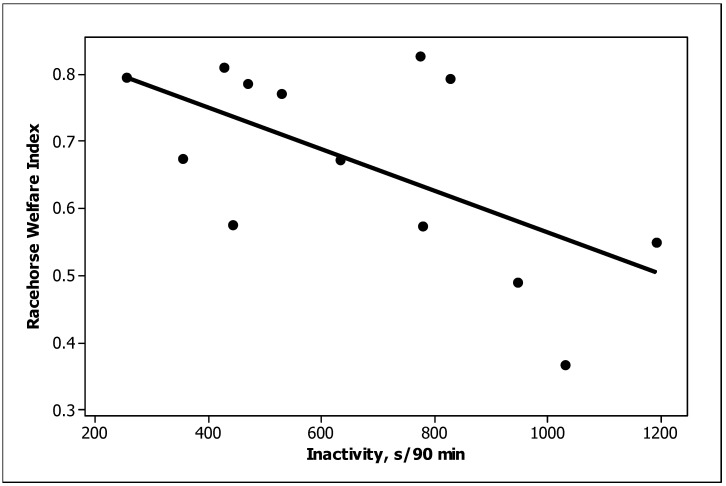
Negative relationship between inactivity time and the TBRWI score.

**Figure 3:**
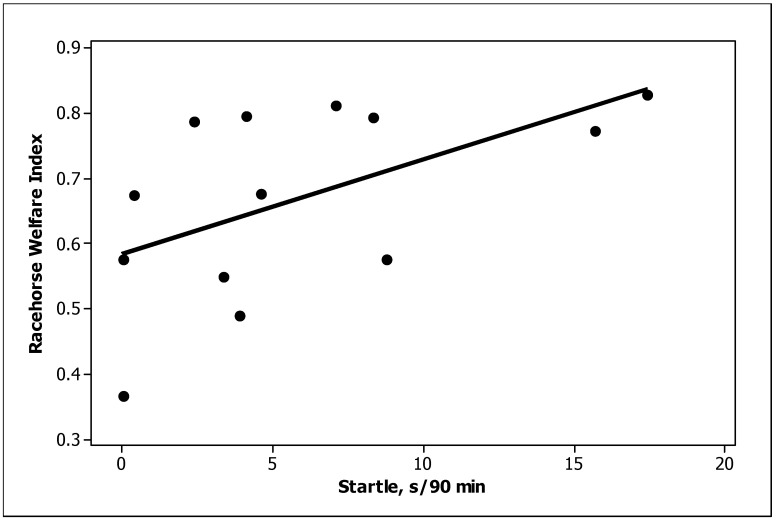
Positive relationship between startle time and the TBRWI score.

**Figure 4:**
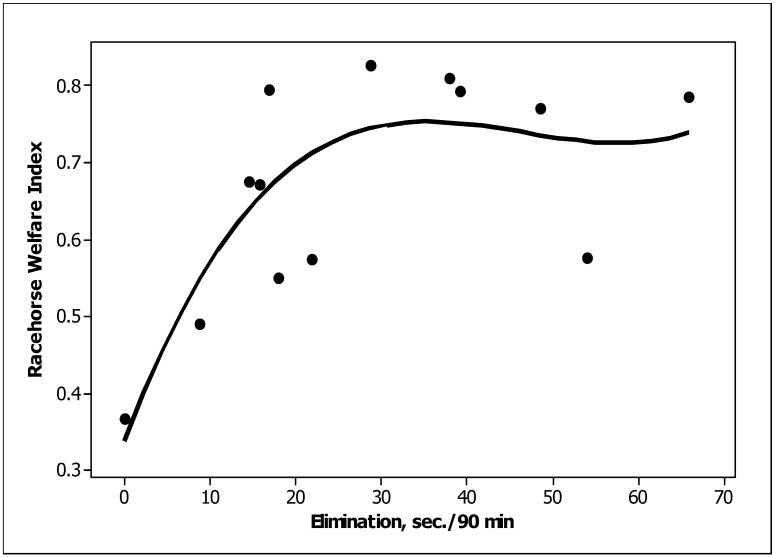
Cubic relationship between elimination time and the TBRWI.

Three behaviour predictors were significantly (*p <* 0.05) related to the TBRWI scores across the thirteen training stables: standing inactive, startle and box walking (Table 5). Standing inactive was negatively related and startle was positively related to the TBRWI score (Figure 2 and Figure 3, respectively). Box walking was negatively related but was considered unreliable because of the major influence of a single stable. A fourth behaviour, elimination, tended to be related (*p =* 0.08); a cubic relationship suggested that the TBRWI and elimination were positively related at low values of both but were unrelated at high values (Figure 4). Only one of the TBRWI issues, nutrition, was correlated with horse behaviour when tested with stepwise regression. Stables with high ratings for the nutrition welfare issue contained horses that spent more time feeding (*p =* 0.003) and less time drinking (*p =* 0.02) (Appendix E).

### 3.2. Study 2—Survey of Trainers Regarding Their Horses’ Performance on the TBRWI

#### 3.2.1. Response Rate

The total number of completed surveys was 58 (email, 32; post, 26). There were only 268 delivered emails, providing 50 responses, 32 of which were complete. The overall response rate for the survey was 11.9%, 3.0% for email and 14.0% for post, with an overall completed response rate for both delivery methods of 4.4%. The actual (including only delivered questionnaires) response rate from delivered emails was 57.5%, and from hard copy, it was 12.1%, with an overall actual response rate of 28.5%. 

#### 3.2.2. Section 1—Demographics 

The majority of the respondents were male (74%) and most were aged over 40 (76%) (Table 6). When asked to indicate the highest level of education achieved, almost half of the respondents had finished education at high school. Nearly all had more than forty-eight months of experience with TBRs, gained in Australia. 

#### 3.2.3. Section 2—Welfare Provision for the Issues in the TBRWI

The responses to the Issue of horsemanship indicated that 81% of the trainers reported that they had staff that were well-trained and experienced, with knowledge of equine behaviour, equine husbandry, management and training, with an affinity and empathy for TBRs (Table 7). Just 5% of the trainers said they had staff that lacked the ability to evaluate health and welfare and frequently resorted to force. The remaining 12% of trainers said approximately half their staff were able to evaluate health and welfare.

Trainers responded to health and disease positively, with 95% saying that they regularly attended to health with the appropriate use of tranquilisers, analgesics and parasitic control, while only 3% of trainers reported that they gave only some attention to health with the occasional use of analgesics, tranquilisers and parasitic medication. The remaining 2% said they attended to health infrequently, and tranquilisers, analgesics and parasitic control medication were used only when absolutely necessary.

A total of 54% of trainers said the education of their TBRs involved regular training from birth to weaning, sales preparation and transporting, riding, track work, barrier habituation and racing for their preparation to commence race training, while a further 41% said their horses came to them with some of the above education. Just 4% said the TBRs had little or no preparation before commencing training.

A gradual turning cambered turf track was the most common type of racetrack, reported to be used by 45% of the trainers. While 13.8% raced their TBR on a tight-turning cambered turf track, and 7% raced them on a gradual-turning cambered synthetic track, only 5% raced them on a tight-turning synthetic track. Other types of racetracks and surfaces that were not described in the questionnaire (e.g., dirt or sand) accounted for 29% of respondents. Trainers complained about the roughness of country tracks. 

Good ventilation, with fans in every stable, and well-ventilated transportation, were reported by 50% of the trainers. In total, 29% said they had some other type of ventilation not listed in the questionnaire; when asked to describe this, they listed walk-in/walk-out stables or openair stables. In total, 17% of trainers said they had stables with only fans at the end of the stable corridors and some ventilation in transport. Only 3% of stables were reported to be of solid construction, with poor ventilation to at least 110 cm, wire mesh above and inadequate ventilation in transport. 

Some 36% of TBR trainers had large stables, 5 × 5 × 6 m, with free use of an attached yard. A further 24% had the same-sized stables but no free use of an attached yard. A total of 14% said they had small stables, 3.6 × 3.6 × 4 m, with no use of an attached yard; 5% had stables of the same size but with free use of an attached yard. A total of 21% of trainers had other types of stabling, mostly walk-in/walk-out buildings with free use of an attached yard or a shelter that was open on three or four sides and situated within a yard.

The trainers’ responses on weaning suggested that a majority, 52%, had little to no knowledge of the weaning process. The isolation of two weanlings together in a stable, allowing for visual and physical contact with neighbouring horses, was the preferred practice for 22% of trainers. In total, 14% of trainers removed one mare at a time from a group of mares and foals until all mares were removed and only foals remained.

Nearly all trainers (90%) reported using skilled drivers who were very experienced in loading and offloading horses. A few (7%) reported using semiskilled drivers who had experience in loading and offloading TBRs, while 2% said they had staff with limited experience. 

Regarding nutrition, most trainers (78%) said that they paid attention to the requirements of individual horses, as determined by age and training, balancing fibre/grain intake and providing access to additional green forage. Some (17%) paid attention to individual horses’ needs but only provided infrequent access to green forage. Just 2% of trainers did not supply any green forage and did not pay attention to individual horses’ requirements. 

A significant proportion of trainers were unaware of what happens to the TBR after retirement from racing (34%), and a similar proportion retired TBRs to equestrian sports (33%). Only a few reported sending horses to the slaughterhouse (1.7%). Some (14%) reported that their horses were given away for other purposes due to poor temperament, insufficient race records for breeding and being unsuitable for equestrian sports. Trainers later identified that this was primarily as companion horses. A total of 17% retired their TBRs to a breeding farm. 

Heat and humidity responses suggested that 50.0% of trainers rarely exposed their TBRs to climatic variation, and their TBRs were acclimatized after transport, with good stable design. Some 38% said that their hoses were sometimes exposed to climatic variation, and 9% said their horses were regularly exposed to climatic variation and had poor stable design and opportunities for acclimatization after transport. 

Regarding whip use, 62% of trainers reported that whips were used on their horses occasionally throughout the race; 21% reported their regular use in the last 120 m of the race if the horses were tired. However, 17% did not use whips; the jockeys rode with “hands and heels” (without using the whip, urging the horse forward with his/her heels and light hand contact on the reins).

Trainers were asked what type of environment the TBRs were kept in and whether the yard design allowed for physical and or visual contact with conspecifics, as well as what type of bedding they used, i.e., wood shavings or straw bedding. Most used wood shavings (76%), and most (62%) of these only offered visual, not physical, contact. Of the remainder, 6% used straw, and 19% used something else, the most commonly cited (7%) being sand, with one trainer using sawdust. 

Concerning gear, i.e. the use of blinkers and tongue ties, 59% of trainers said they used both, while 24% used neither. Blinkers but not tongue ties were used by 16%, with 2% of trainers using tongue ties but not blinkers.

#### 3.2.4. TBRWI Scores

**Figure 5:**
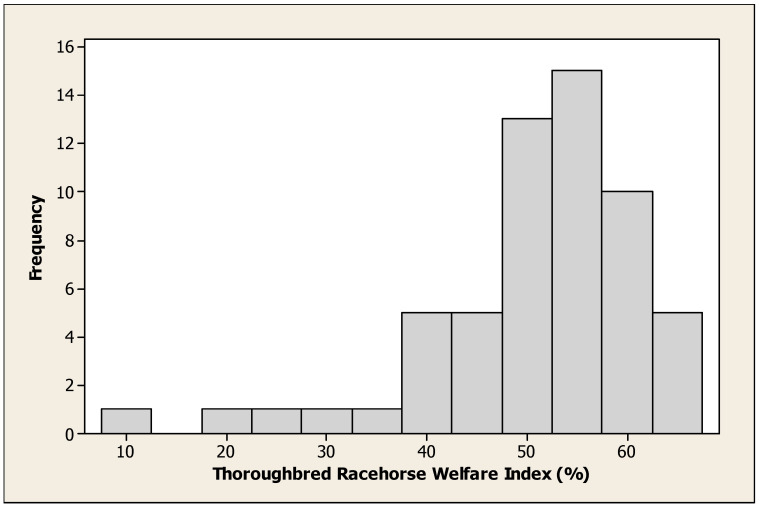
Distribution of TBRWI scores (%) for the 58 trainers responding to the survey in Study 2.

The TBRWI scores determined from the 58 trainers’ responses were not normally distributed (Table 8, Figure 5). The mean score was 50.75%, the SEM was 1.35, the median was 52.95% and the maximum and minimum scores were 63.6 and 11.6%, respectively.

**Figure 6:**
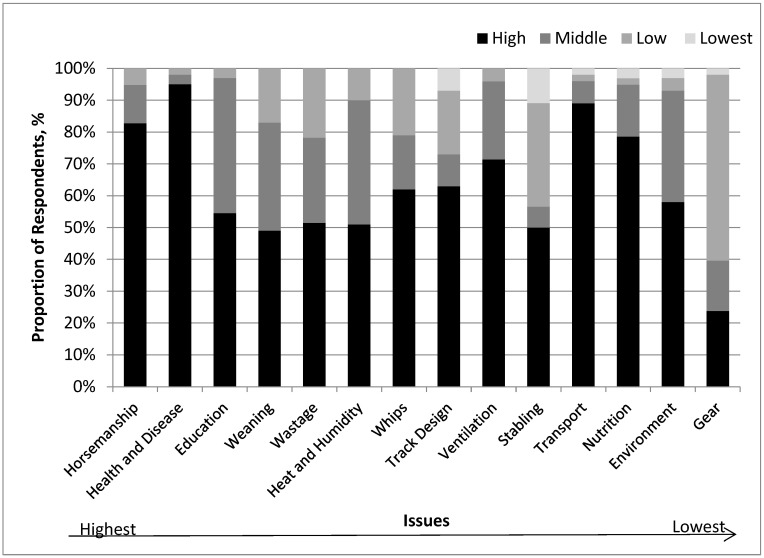
The percentage of TBR trainers selecting each issue level (lowest, low, middle, high) in Study 2, with issues ordered from highest (lefthand side) to lowest (righthand side) importance.

In Figure 6, we depict the summary information for the welfare issues—arranged from the most important on the left to the least important on the right—in order to determine which issues would best be targeted for welfare improvement (those towards the left side and where few trainers performed at a high level). Of the issues, the first two (from the left) have trainers performing at good levels, but the next three have only about 50% performing at the top level. Scores of 81% and 95% were achieved for the top level in horsemanship and health and disease, the first two highly scored issues indicating a high level of welfare. Those issues that were important but had a limited number of top achievers were weaning, wastage, heat and humidity, stabling and education of the TBR, with only about 50% performing at the top level, indicating there is a need for improvement in these welfare issues. Towards the lower level of importance, nutrition scored highly (slightly below 80%, achieving the top level). Transport was also towards the lower level of importance for the issues, but had just under 90% achieving the top level. Towards the middle of the scale were the issues track design and surface, whips, ventilation and environment, all with above 60% achieving the top level, with the exception of environment, which scored just under 60%, indicating that there is a need for improvement in this issue. Gear had the lowest score of 24% achieving the top level, but it was not rated by the industry as an important issue.

**Figure 7:**
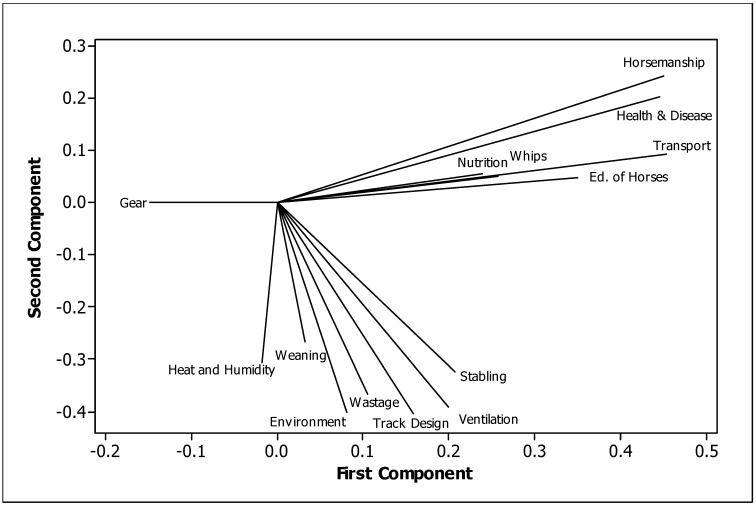
Relationship between first and second principal components for TBRWI issues.

In the PCR of TBRWI issues, the five components with eigenvalues of more than 1.0 explained 63% of the variance. The loading plot for the first two, which explained 20 and 15% of the variance, respectively, is illustrated in Figure 7. These had eigenvalues of 2.82 and 2.08, respectively. This showed that the use of gear was antagonistically related to animal-related issues in the first principal component, which, in turn, were antagonistically related to facility-related issues, including ventilation, stabling, track design, environment, heat and humidity, etc.

#### 3.2.5. Gender Differences in Responses to the Trainer Survey 

Of the 14 issues tested, 5 had significant or close-to-significant gender differences:

In track design and surface, after combining levels 2, 3 and 4, male respondents (M) were more likely than female respondents (F) to say that their horses raced on other types of tracks (gradual turning turf: M = 17, F = 9; gradual or tight turf or synthetic: M = 10, F = 5; other: M = 16, F = 1) (Χ^2^ = 6.1, *p =* 0.048).

For weaning levels, 2/3 and 4/5 were combined. Compared with men, women were relatively more likely to say that the most common weaning practice was one or two weanlings in a stable (M = 8, F = 8) rather than the removal of one mare at a time from a paddock (M = 7, F = 1) or unknown/other (M = 28, F = 6) (Χ^2^ = 6.4, *p =* 0.041).

After combining nutrition levels 2, 3 and 4, men were more likely than women to say that their horses’ nutritional needs were tailored to their requirements, with access to supplements and green forage (M 36, F 9), whereas women were relatively more likely to report a less satisfactory standard of nutrition (M 7, F 6) (Χ^2^ = 3.3, *p =* 0.068). 

When levels 2, 3 and 4 were combined in wastage, men were most likely to say they did not know what happened to their horses when they finished racing (M = 20, F = 0), whereas women were relatively more likely to say that the horse was retired to a breeding farm (M = 5, F = 5) or that the horse was used for equestrian sports, given away or slaughtered (M = 18, F = 10) (Χ^2^ = 15.9, *p* < 0.001). 

In relation to the education of the TBR, women tended to be more likely than men to say that horses received some or no education, whereas men were more likely to say that they received regular education (Χ^2^ = 3.7, *p =* 0.056).

There were no differences between men or women for horsemanship, health and disease, ventilation, stabling, transport, heat and humidity, whips, environment or gear.

## 4. Discussion 

### 4.1. Study 1—Horse Behaviour in Training Stables and Its Relation to TBR Welfare Index Scores

Horses spent most of their time feeding, standing, looking and standing inactive. Looking was the most common of TBRs’ responses to external stimuli; the head would be orientated towards the object or animal with an elevated, rigid neck, with ears erect and slightly dilated nostrils [17,18]. 

The most variable behaviours in our study (Table 2) were rocking, chewing objects, inactivity, resting, elimination and drinking, which can all be associated with stress [16,45]. The time budget of the Thoroughbred racehorse (TBR) is influenced by the management system, but they often only spend 10 [46] to 40% [38] of the day consuming their ration of concentrates and hay, in contrast to free-ranging or feral horses, who typically spend 70% of the day foraging [17,47,48,49]. Horses in our study spent 32% of their daytime recording period feeding, which suggests that feeding of forage was more adequate than in Marsden’s study [46]. However, horses in our study spent over a third of their time standing inactive and looking. The duration of standing inactive was negatively related to the TBRWI scores for the horses, which emphasizes the importance of providing good welfare resources and demonstrates the ability of this component of the TBRWI to monitor welfare effectively. When standing inactive, TBRs are lethargic, their heads lowered and ears upright. An abnormal amount of time spent in unusual postures may be associated with physical discomfort and may indicate problems of physical illness, weakness or social stress [25,50], i.e., poor welfare [18,23,24,26]. Apart from standing inactive, startle and box walking were correlated with the TBRWI.

The similar relationship to that observed between standing inactive and TBRWI scores seen with box walking demonstrates the importance to welfare of this variable. Furthermore, box walking was negatively related to feeding and drinking, suggesting that horses with inadequate feeding and drinking resort to box walking to occupy their time. Box walking involves the horse moving in a repetitive, aimless pattern [10,36], which can form a figure 8, a circle or along a wall in the stable, or moving rhythmically from side to side, i.e. weaving [37]. In our study, box walking increased with the age of the horse, which may demonstrate that frustration levels increase over time. 

Oral stereotypies also occurred during the stable observations, the most common being windsucking and crib-biting. Both behaviours involve tensing the neck muscles and taking a breath, with crib biters (but not windsuckers) grasping a solid object [10,18]. Chewing objects is similar to windsucking/crib-biting and can damage stable fittings, especially when wooden. Pieces of wood or splinters may be broken away and ingested, subsequently harming the TBR [10,18]. Sham chewing was also observed when a TBR’s mouth was empty while it repetitively moved the jaw and tongue [18,35]. Repetitive licking of the sides of buckets, walls, floors and door latches also occurred without a nutritional purpose attached to this behaviour [10,35]. Yawning was observed during long inhalations, with the TBR’s mouth wide open, at times moving its jaws from side to side [18].

The startle response was more common in racehorses with a high TBRWI. Characterised by a rapid behavioural reaction to a sudden stimulus and associated with neurohormonal changes related to stress [51], it was unexpected that this would be associated with good welfare. However, it may indicate that the horses are responding well to external stimuli. The TBR’s head is typically up with erect ears and a tense neck and back, and it may rush away or turn suddenly. A similar reaction in a feral horse would evoke a swift departure (positive welfare, being lifesaving), but in a stabled TBR, the most common reaction is just a tense neck and back.

The tendency for elimination to be related to the TBRWI in a cubic relationship may be because stressful conditions are known to be a trigger for elimination; e.g., Ecke and Hodgson [52] found transportation was a cause of the development of colitis. TBRs who experience the stress of environmental changes and alteration to their workloads are at a high risk of gastrointestinal dysfunction [13,26,53,54,55]. Elimination and rubbing were both increased in males, evidence of their tendency to want to mark their territory in a latrine. Elimination includes urination and defecation, both of which are sexually dimorphic [18]. The distinct male and female forms of defecation [17,18] were observed, as males tended to make faeces piles and urinate in one area of the stable. Females defecated and urinated indiscriminately within the stable. The amount and frequency of elimination also vary according to the amount of food and water consumed [18]. Elimination was associated with some stereotypic behaviour, notably rocking, which suggests that territorial behaviour is one motivating force for some of the stereotypies we observed. When horses are fed a high grain diet, with little roughage, elimination is limited because of the reduction in the total bulk consumed in 24 h, thus explaining the positive cubic relationship between stables with a low TBRWI and elimination.

### 4.2. Study 2—Survey of Trainers Regarding Their Horses’ Performance on the TBRWI

The rate of response from the trainers to delivered invitations (29%) was better than the response rate from trainers for some other TBR surveys [56,57]. The town and suburban addresses and phone numbers of trainers were relatively easy to obtain, displayed in racing journals that list trainers’ licenses and registrations. However, many of the street addresses proved to be invalid, especially for those hard copies sent to country areas, and they were consequently returned unopened. The racing industry does not list the email addresses of racing trainers by state, and a method of listing trainers’ email addresses for each state would assist in obtaining data.

Assessing the welfare of TBRs is more complex than farm and zoo animals, given the husbandry and the work requirements of the TBR. The welfare of farm animals can be partly assessed by their production of milk and eggs [58] and reproduction rate [59]. The welfare of captive wild animals in zoos is now often assessed by the management and environment that best equates with their respective wild environments. Such parameters are of limited relevance when assessing the welfare of Thoroughbred racehorses due to the restricted housing [46,60] and feeding programs [61,62,63]. These management systems are used because TBRs require individual management and high protein rations for peak performance.

The assessment of multiple contributing parameters in developing a Thoroughbred racehorse welfare index, as in the present study, is one method of improving husbandry and welfare, especially when combined with behavioural parameters. Figure 7 demonstrates differences in responses to animal and environment-based issues. Here, the use of gear was antagonistically linked to animal-related issues in the first principal component, which in turn was antagonistically related to facility-related issues. 

#### 4.2.1. Trainer Performance on Individual Issues

The questionnaire attempted to address the main welfare issues regarding the care of TBRs in race training, highlighting the need for regular exercise, mental stimulation and the provision of companions [45,53,64,65,66,67,68] whilst also ensuring “that their natural capabilities are able to cope with the task of being a racehorse” [45].

The highest level of horsemanship was chosen by 81% of trainers (Figure 6). Only 5% of trainers admitted their staff were inexperienced and lacking in training and did not employ knowledge of equine behaviour in management and training. For instance, knowledge of management and training are important factors in preventing the occurrence of learned helplessness (standing inactive) in TBRs, an abnormal behaviour, which results in the loss of motivation, anhedonia and stomach ulcers, as well as weight loss [18,26,69]. Trainers were likely to have been reluctant to admit to any shortcomings in their staff; however, using the index in the field would probably not rely on self-assessment.

The health and disease factor was apparently well managed by the trainers, with a score of 95% demonstrating the importance placed by trainers in ensuring the physical capabilities of the TBR and giving it every chance to perform at its optimum level.

The education of TBRs received a score of 54% in the trainer survey. This relatively low score indicates there is a need for greater education of the TBR before it goes to the trainer to commence race training. The education of the TBR is an ongoing process from birth. Waran et al. [68] advocate the early handling and education of the foal to adulthood in order to prevent the development of problem behaviours, provoked by fearful situations [28,70,71]. Early education and handling provide the basis for subsequent “foundation training” [1,28,72].

The track design and surface factor was scored at the highest level by 63% of the trainers. The high score may be due to their awareness of the number of injuries sustained by TBRs on the racecourse [2,73,74,75], with poorly designed tracks and neglected surfaces being a major welfare concern for TBRs [25,74]. Rough country tracks create a major risk during drought conditions and demonstrate the limitations of financial and material resources. There is a need to maintain the moisture content of track surfaces during dry periods.

Ventilation was rated at the highest level by 70% of the trainers. Poor ventilation adversely affects the respiratory system of the TBR [13,76,77], sometimes leading to obstructive pulmonary disease [78]. In situations with poor ventilation, a horse will stand for longer periods of time than is normal, indicating stress [38].

Stable design was rated at the highest level by just 46% of the trainers. Design is changing in Australia due to the upgrading of city and metropolitan racecourses. Many of the older racecourses and sale centres are now surrounded by suburbia, which increases the pressure on space available for new and adequately designed stabling [12,17,78,79,80,81]. Improved stable design will aid in the control of climatic variation, e.g., tie-up stalls at racecourses should not be situated so the TBR stands in the sun all day [82]. Many stables are too small [5], with the roof height rarely considered [7].

The Australian Horse Welfare Protocol [83] states that the stable size “should not be less than 12 m^2^ with a ceiling height not less than 2.4 m”. Occupational health and safety regulations differ between Australian states and are based on minimum requirements that do not relate the size of the TBR to the size of the stable, nor do they include the roof height [84]. Sainsbury [5] advocates stables measuring 4 × 4 m, with a ceiling height at the eaves of 3 m rising to a roof ridge of 8 m. Our survey found that 36% of trainers in Australia used stables with a floor size of approximately 5 × 5 m, with a ceiling height of approximately 6 m and free use of an attached yard, while only 5% of stables were approximately 3.6 × 3.6 m, with a ceiling height of approximately 4 m and free use of an attached yard.

Weaning was rated at the highest level by just 49% of the trainers, with 52.0% of trainers having no knowledge of how their TBRs were weaned. The management of foals at weaning time is crucial to prevent oral stereotypies from developing. The gradual removal of one mare at a time from a group of mares and foals until no mares remain aids in the prevention of oral stereotypies [79,80,85]. Foals that are paddock-weaned in small groups had similar time budgets to feral horses [23]. A fibre-rich pasture supplement at weaning time will not only reduce stereotypic behaviour [63] but will also prevent the development of gastrointestinal ulcers [54,79,80,85,86].

Many trainers, especially male trainers, were unaware of what happens to TBRs when they finish racing. This suggests more empathy in female trainers. Rehoming is a desirable option, with many TBRs sold for a small amount or given away for equestrian sports [87]. Many TBRs never reach the racecourse due to injury, an intractable temperament or being too slow [4,74,88]. As the trainer rarely owns the TBR, it is the owner who makes the final decision [88,89].When injuries prevent further use [90], the temperament is unsuitable or their breeding is considered below the commercial level, they may be destroyed. 

Transport was scored at the top level by 90% of trainers, indicating the importance placed by trainers on this management issue. Nutrition was recognised by 78% of trainers as important to the well-being of TBRs since it was achieved at the highest level [91]. Nutrition was the only issue to correlate with the behaviours observed in our stable study (feeding and drinking). Thoroughbred horse trainers are aware of the importance of an optimum condition score by monitoring body weight, which is a component of physical welfare [61,62,92]. To win races, which is the aim of racehorse trainers worldwide [88,89,93], TBRs must be neither thin nor overweight [62,92]. Although the diet may be carefully balanced and formulated to meet all the dietary needs, it may not meet the horse’s behavioural needs [16,17,94]. In the training management system, discrete meals are delivered two or three times a day as opposed to the continuous grazing behaviour of the feral horse [48,94,95,96,97]. In our study of stable behaviour, TBRs only spent 32% of the recorded period, which was at key feeding times, actually feeding. The decreased gastrointestinal function that ensues [54] can lead to problems in the digestive system, resulting in colic, laminitis and gastric ulcers [98]. Gastric ulcer pain is a factor in the development of a TBR’s stereotypic behaviour [54,85,91,99]. Thus, to meet the TBR’s nutritional and behavioural needs, a diet of balanced nutrition with a variety of forage is important in maintaining adequate gastrointestinal function [54].

Heat stress management was scored at the highest level by 50% of trainers. Most TBRs have a wide tolerance for temperature variation; equines can cope with a temperature range varying from freezing to high heat in desert conditions, and relative humidity can be as high as 100% [17]. Competition horses travelling from temperate climates to hot-humid countries require a period of about 10–14 days to acclimatize [82]. Equestrian Australia’s Hot Weather Policy covers the care of TBRs racing in extreme heat, i.e., when ambient the temperature is 35 °C or above and the wet bulb global temperature (WBGT) in the shade is 26 °C or above [82]. The policy suggests that horses should be stabled out of the sun. Racecourse designs should take into account the movement of the sun throughout the year when designing day stalls [83].

A total of 62% of trainers scored the use of whips as “occasional, throughout the race”, while 21% admitted that their horses were whipped regularly in the last 100 m of the race, even though they were tired. Queensland’s whip rules allow jockeys to use the whip as often as they like in the last 100 m of the race, thus allowing the TBR to be struck as many as 13 consecutive times [82]. The padded whip is designed so that a portion is not padded, and where the different sections meet, there is a hard knot. Research by McGreevy and Evans [100] found horses were struck with this part of the whip on 64% of occasions when the whip was used. The Australian Racing Industry (ARI) is a signatory to international guidelines preventing TBRs from being whipped on the flank and abdomen, but TBRs are usually hit on the flank and abdomen and sometimes hit on the head [101]. The horse evolved as a prey animal [102,103,104] with stress-induced analgesia, i.e., the ability to mask pain. Newby [105] believes that “the TBR’s had learnt to tolerate the whip”, which can be described as learned helplessness or standing inactive [18,25]. Excessive whipping of an exhausted horse, when it no longer can respond, may cause “learned helplessness” or “standing inactive” [18]. There is evidence that the rules regarding whip use are frequently breached [106]. Furthermore, the jockey’s fine is small compared with the winning horse’s prize money [107]. The Chief Executive of the Australian Racing Board Ltd (ARBL) has indicated that “The ARBL would be fully supportive if stewards reversed the result of a race over (the) use of the whip if circumstances demanded it” [108]. This may be one way of preventing the overuse of the whip. At a later interview, the then-Chief Executive of the ARBL (Peter McGauran) said that “the industry would further restrict the use of the whip or do away with it completely if there was evidence that repeated whipping inflicted pain’ [109]. McGreevy and McLean [110] believe it is “important that the whip is not used to deliver sharp punitive pain”. Use of the whip can be seen as punishment (for not running fast enough). Both Mills [111] and McGreevy and McLean [112] believe punishment “is best avoided, as it presents a range of problems that amount to abuse”.

An environment that included physical and visual contact with other TBRs was selected by just 29% of trainers. Wood shavings and a stable yard allowing only visual contact was the most popular stabling system, with 47% of the trainers selecting this option (Table 7). Physical and visual contact is important for the well-being of TBRs [30,61,113], yet few trainers provided physical contact.

Wood shavings used by most trainers are more absorbent than straw, and they are rarely eaten by the TBR, but straw bedding is eaten [12,30,62,114,115]. This may explain why horses that were bedded on straw had fewer oral stereotypies than horses bedded on shavings [10,15,115]. Bed eating is a redirected behaviour [16] that involves “the ingestion of bedding substrates such as straw, paper or shavings” [115]. This behaviour is more common in horses that do not have access to high-fibre forages such as hay, and it is more common in horses that are bedded on straw than those on rubber, paper or shavings [115]. McGreevy et al. [15] noted that “horses on straw also have fewer oral stereotypies such as crib biting”.

Trainers did not offer physical contact with other horses in the stable yard. Some stable designs [5,13] have full-height walls, justified by the need to prevent cross-infection of airborne pathogens [13,77,116]. Trainers fear that physical contact may result in injury; thus, TBRs are kept in individual stables [13].

Few trainers reported the highest score for the use of gear, i.e., no blinkers or tongue ties. The use of tongue ties is approved by the RAL, but it is there to protect the horse from swallowing its tongue or the dorsal displacement of its soft palate, which interferes with breathing. These are safety issues, and its use alone was reported by 2% of trainers, while blinkers alone were used by 15.5% [3]. Tongue ties and blinkers were used by most (59%) trainers, mainly for the safety of all concerned [28,110]. They are approved by the RAL and can be used singularly or in combination.

#### 4.2.2. Gender Effects

Men were more likely to lack clarity in their responses than women; for instance, in education, the men were more likely to say that their TBRs received regular education, while women were more inclined to answer the question in a straightforward manner, admitting their TBRs received little education or none at all. More favourable responses for nutrition were given by men. Women demonstrated a more sympathetic approach to the welfare of TBRs than men did, demonstrating empathy for the TBR, whereas men tended to ignore situations over which they had little control, with their responses being more guarded for the wastage, weaning, track design and surface issues.

### 4.3. Implementing the TBRWI

We believe our research methodologies are the most specific to the development of a welfare index for Thoroughbred Racehorses to date. Tadich et al.’s study [10] of Chilean racehorses was concerned with estimating the prevalence of stereotypic and other behaviours in Chilean Thoroughbred racehorses by directly observing and examining their associations with biological characteristics and management practices. A questionnaire was also administered to handlers. Collins et al. [9] used a three-round, web-based Policy Delphi method to canvass opinions on the perceived most significant equine welfare issues. The aim of Petersen et al.’s [6] study of warmblood riding school horses in Schleswig-Holstein, Germany, was to gain insight into the practical experience of horse keeping in Schleswig-Holstein. The concept of the “evaluation of livery stables with regard to animal welfare” was developed by Beyer [117] to determine high-risk elements in livery stables, e.g., stabling, environment, ammonia concentration, health and the behavioural disturbances of individual horses using direct observation and a point system. None of the above research studies, though concerned with equine welfare, developed a system of welfare assessment, and as such are not comparable to a TBRWI, which is a uniform welfare method capable of measuring husbandry within the Thoroughbred racing industry.

This TBRWI could potentially detect those racetracks in Australia that are most in need of improvement, as they are often outside metropolitan areas and have limited available funds. Racetrack designs and surfaces are sometimes poor and have limited surface care, with many racecourses being too small with tight turns and insufficient camber [118], even though land availability is seldom a problem in country areas. Small country racecourses are often installed without consideration of the dangers involved or the subsequent wastage of TBRs and, sometimes, danger to their riders when racing on small, tight-turning tracks. This type of track can lead to dorsal metacarpal disease in TBRs’ front cannon bones [2,73,74,88], sometimes ending in fracture. The cost of track maintenance and surface design should be considered if the wastage of TBRs is to be reduced. Most injuries occur at training [2,73,74,88]. 

New stabling facilities are under construction on many of the older racecourses in Australia, some with physical and visual contact for all TBRs. The optimum dimensions of these stables are not known, and little research has been undertaken. Sainsbury [5] considers stabling in the Northern Hemisphere, however there is no suitable reference material for the construction of stabling in Australia and other countries that do not experience severe winters (below freezing) or tropical conditions. 

Racing boards are responsible for the design of racetracks and surface care. “The Australian racing industry has not adopted a rigorous approach in identifying the causes of wastage associated with track injuries within the racing industry” [74]. It has been reported that Australian racehorses have the highest incidence of shin soreness in the world [118]. To counter claims of unacceptable injury rates, racing boards can implement improvements to track designs and surface care and introduce policies that address areas of welfare concern, identified by the TBRWI. Central to the issue of racetrack design and surface is the improvement of racetracks with small radius bends and track banking, as well as the softening of hard track surfaces [118]. The TBRWI has the ability to highlight the importance of safety issues in racetrack design in order to lower the incidence of track-related injuries of both horses and jockeys in Australia and in countries with similar climates whose racetracks have hard surfaces. 

The TBRWI could aid governments in formulating codes of practice (embodying welfare issues) and in legislating equine welfare. The use of the index is enhanced by the easy checklist format of the TBRWI in assessing welfare standards within the racing industry. This index is also unambiguous and ensures common interpretations across assessors, as well as being non-invasive. 

A need to change weaning practices in Australia could be identified by the TBRWI’s weaning methodology [23,119]. New methods involve removing the mare, the dam of the oldest foal, from a group of mares and foals, continuing to do so until all wet mares are removed from the paddock (or field), leaving an older equine as a stabilising influence (the nanny). 

Gear could be deleted from the list of welfare issues, bringing the number to 13 issues instead of the original 14 identified at the stakeholder meeting [40]. Gear was consistently rated as relatively unimportant both in the survey of MacTaggart and Phillips [40] and in the trainer survey reported in this paper. 

#### Limitations of the Study

The interpretation of some issues may have caused confusion, e.g., environment, when visual contact was preferred above visual and physical contact. The respondent may have surmised that having physical contact meant more than one TBR would be in the stable at any time. Physical contact is possible between horses in adjoining stables when partitions are not solid but made of bars, slats or heavy wire and wide enough to allow horses to sniff and or touch one another. 

Trainers may have been reluctant to admit to any shortcomings in their facilities or their staff, but, as mentioned previously, the field use of the index is unlikely to be self-reported. They may also have been unaware of the weaning method used for their TBRs.

### 4.4. Conclusions

Using the TBRWI, the first part of this study identified that several key behaviours were related to performance on the index across a range of training stables. In the second part, we identified that a range of performance levels was achieved by trainers and determined which might be most successfully targeted for improvement. Although horsemanship and health and disease control were mainly claimed to be at the highest level, there was significant room for improvement in the education of the horse; weaning practices; wastage and managing heat and humidity; and track design and surface. Lesser gains could be achieved by improving the rules of whip and gear use, but the latter was not seen to be sufficiently important for welfare to be included in the TBRWI.

## Figures and Tables

**Table 1:** Definitions of behaviours in observations of Thoroughbred racehorses (*Equus caballus*).

Behaviour	Description
Maintenance	Daily activities that keep the animal alive and well [18].
Feeding	The ingestion of feed. TBRs anticipate the arrival of their feed with an increase in activity and sometimes perform stereotypic behaviour; see door banging [18,19].
Drinking	The imbibing of water. Drinking usually occurs at the same time as feeding; however, drinking rhythms may vary [19].
Elimination	The voiding of faeces or urine. The behaviour is sexually dimorphic, with females eliminating at random with no specific attention to their faeces or urine and stallions and geldings tending to pile faeces and urinate in the same area in the stable [18].
Lying	Lying down with head up or with head and legs outstretched [18,20]. TBRs rest or sleep in either lateral or sternal recumbency. Lying occurs after morning exercise and/or feeding and on days after racing [21].
Standing resting	Standing in a relaxed posture, head slightly lowered and eyes partly closed, usually one hind leg flexed, ears rotated laterally [17,18]. Standing resting is characterised by a general lack of attention and inactivity and is a transition period between wakefulness and sleep.
Standing sleeping	Standing sleep with closed eyes and head lower than the back [18]. At such times, the equine body is supported by the equine stay apparatus [22].
Standing Inactive	Dull and quiet manner and/or lethargic, associated with “physical illness, weakness or severe social stress” [18,23,24,25,26], or the TBR may be exhausted from recent racing [21].
Rubbing	“Licking, biting, rubbing and scratching part of the body” [18] (McDonnell, 2003). Often differentiated into autogrooming (self-grooming) and allogrooming (two TBRs groom each other).
Social Communication	Communication is both visual (using signals made by postures and expressions of the ear, tail, face and mouth) [19] and auditory, i.e., vocalisations (whinnies, neighs, snorts, squeals and grunts) and sounds, i.e., hooves (pawing and stamping), as well as chemical cues [18].
Play	Object play [18] involves the manipulation and contact of an object that may be inanimate or animate. The management restrictions of stabled TBRs prevent most types of play.
Sniffing	Drawing air in through the nostrils [18,27]; the TBRs frequently place their noses close to the object of interest and sniff.
Aggression	Aggression involves the extension of the head and neck towards an individual with flattened ears, threats and bites [28]. Recognised by agonistic signalling [29]. Occurs mostly at feed time between conspecifics when indicating ownership of their feed [30]. Stabled TBRs sometimes demonstrated ownership of space [30] and aggression towards humans who failed to respond to subtle threat signals [31].
Vocalisation	Sounds produced by TBRs via their vocal tracts [32]. Snorts and snickers are from the nose, with mouth closed, while a squeal is defensive and, like the neigh or whinny, is produced with the mouth open [19]. Neighing or whinnying was frequently heard either in greeting or indicating anxiety, i.e., when separated from conspecifics. Snorts and sneezing were frequently heard when TBRs exercised.
Walking	A voluntary symmetrical gait with 2, 3, or 4 legs supporting the TBR. Observed as a slow, four-beat forward movement with an equal interval of time between footfalls [33]. The most commonly observed locomotion occurred between water and feeding stations or between a yard and the stable when free use of a yard was available [17].
Oral stereotypic behaviours	Repetitive, aimless behaviour performed with the mouth with no obvious goal or function [34]. Such behaviour can interfere with time spent resting or feeding [16].
Windsucking/crib- biting	Crib-biting is a sudden intake of air as the TBR repeatedly grasps a fixed object, tensing the neck muscles and, at the same time, pulling back as air is sucked into the oesophagus [28]. Wind sucking does not involve grasping but in all other aspects is the same as crib-biting [10,18].
Chewing objects	Chewing is a “side to side grinding motion of upper and lower jaw on an object in the mouth” [18]. TBRs occasionally chewed wooden objects such as the top of the stable door or the divider rails of the stable.
Sham chewing	Sham chewing occurs with an empty mouth, moving the jaw or tongue in a repetitive movement [18,35].
Licking	Licking occurs when the tongue makes contact with an object [18]. Some TBRs lick the sides of their feeders and others lick door handles and wooden stable divider walls [10,35].
Yawning	Yawning occurs by opening the mouth wide and slowly inhaling air, sometimes moving the jaws from side to side [18].
Locomotor stereotypic behaviours	Abnormal repetitive behaviour patterns that are performed with no obvious goal or function. TBRs move in a repetitive pattern around the stable, swaying their body from side to side, moving their head in a repetitive manner, stomping one or more legs or wall/door kicking and/or banging. This repetitive behaviour can interfere with feeding time and/or resting [16].
Box walking	A stylized, repetitive locomotion performed with any gait, usually along a perimeter [18]. TBRs walk repetitively in a pattern, usually along one wall of the stable with the head high and the neck drawn back into the shoulders, before turning to move in the opposite direction, in the same intense manner. Other TBRs use circular or figure of eight patterns. There is no apparent purpose as to where the TBR is going [10,36].
Rocking	Rocking is a rhythmic weaving movement involving repeatedly moving the body, head, neck, shoulders and forelimbs from side to side, sometimes including the hindlimbs as well [37]. Stables with preventative grills fitted to openings, such as doors or windows, prevent most weaving, but treading may then take its place, i.e., moving limbs up and down on the same spot [38].
Door banging	Repeatedly knocking on the door or walls with a forehoof [36,37]. Feeding time is often preceded by repeated door banging.
Head tossing	Repeatedly moving the head up and down, usually ending in a jerky motion [10,39].
Pawing	Continual movement of one foreleg up and down in a digging motion, sometimes directed towards the ground, sometimes directed towards the stable door [10,18].
Response to external stimuli	Sometimes referred to as stand-stare, standing alert or standing attentive. [18].
Looking	Standing motionless and rigid for long periods with ears stiffly upright and forward and nostrils slightly dilated [17]. Also sometimes described as standing alert [18].
Startle	Head held erect with upright ears and oriented towards the external stimulus. It conveys a reaction that is essentially a surprised expression, as though suddenly shocked or frightened. Sometimes TBRs rush away or turn suddenly, with a tense neck and back.

**Table 2:** Mean values for behaviours: percent of time spent by each horse, in descending order, together with the SEM and CV of each behaviour.

Behaviour	Mean, % Time	SEM	Coefficient of Variation, %
Feeding	32.00	0.162	1.87
Standing sleeping	21.60	0.088	1.02
Looking	20.05	0.088	1.02
Standing inactive	16.30	0.244	2.82
Walk	4.55	0.085	0.98
Drinking	1.16	0.191	2.20
Licking	1.01	0.208	2.39
Elimination	0.70	0.80	2.00
Standing resting	0.56	0.247	2.85
Rubbing	0.32	0.159	1.83
Sniffing	0.26	0.151	1.74
Rocking	0.25	0.211	2.43
Chew object	0.23	0.185	2.14
Out of sight	0.21	0.247	2.85
Aggression	0.18	0.14	1.62
Headtoss	0.13	0.13	1.53
Windsucking/crib-biting	0.10	0.15	1.75
Lying	0.09	0.16	1.90
Startle	0.08	0.12	1.40
Pawing	0.05	0.09	1.09
Play	0.05	0.01	1.34
Box walk	0.04	0.08	1.02
Vocal	0.03	0.07	0.90
Yawn	0.02	0.07	0.80
Sham chewing	0.01	0.06	0.71
Door banging	0.01	0.03	0.40

**Table 3:** Eight principal components (PC) for behaviours of TBRs in stables (*n* = 133).

Variable	PC1	PC2	PC3	PC4	PC5	PC6	PC7	PC8
Drink	−0.18	0.41	0.06	0.13	−0.19	−0.01	0.02	−0.18
Feeding	−0.22	0.38	0.20	−0.28	−0.09	0.09	0.05	0.07
Elim	0.30	−0.01	0.08	0.18	−0.19	0.08	0.07	0.16
Lying	0.03	0.06	−0.24	−0.38	−0.29	0.05	−0.20	0.16
St. resting	0.10	0.34	−0.20	0.31	0.19	−0.02	0.09	−0.18
St. inactive	0.10	0.18	−0.31	0.08	−0.23	0.11	0.31	0.24
St. sleep	0.23	0.14	−0.35	−0.13	−0.02	−0.11	0.13	0.10
Rubbing	0.27	0.14	0.19	0.12	0.09	−0.03	−0.23	0.04
Aggress	0.24	0.12	0.21	0.15	0.01	0.16	0.14	0.44
Vocal	0.23	0.04	−0.13	−0.25	0.23	0.31	−0.03	−0.24
Sniffing	0.21	0.28	0.26	0.17	0.13	0.10	0.08	0.16
Boxwalk	0.25	−0.17	−0.04	−0.21	−0.02	0.33	0.18	−0.20
Walk	0.23	−0.24	−0.04	−0.12	−0.15	−0.22	−0.21	−0.11
Play	0.25	0.16	0.15	−0.06	−0.22	0.33	−0.11	−0.28
Windsuck/ crib bite	0.06	−0.01	−0.40	0.32	0.03	0.05	−0.00	0.01
Chew ob	0.14	0.13	−0.39	0.20	−0.09	−0.22	−0.36	−0.02
Shamchew	0.17	0.19	0.04	−0.17	−0.40	−0.08	−0.20	−0.03
Licking	0.21	−0.09	0.15	−0.03	−0.33	−0.21	0.13	0.16
Yawn	0.12	0.08	0.24	−0.08	0.08	−0.55	−0.06	0.04
Weave	0.18	−0.45	0.09	0.27	−0.15	0.09	0.06	0.07
Doorbang	0.10	−0.03	0.07	0.01	−0.20	−0.21	0.52	−0.47
Headtoss	0.26	−0.02	−0.01	−0.26	0.41	0.02	−0.09	0.17
Paw	0.18	0.03	0.18	0.25	0.03	0.03	−0.32	−0.33
Look	0.26	0.11	−0.03	−0.16	0.11	−0.12	0.04	−0.08
Startle	0.17	0.07	−0.06	−0.13	0.25	−0.30	0.30	−0.05

**Table 4:** Significant effects of gender on horse behaviour.

	Female	Male	SED	*p* Value
Elimination (log_10_ % time)	0.72	1.04	0.097	0.03
(% time)	5.3	11.0		
Rubbing (log_10_ % time)	0.47	0.74	0.089	0.05
(% time)	2.9	5.5		

**Table 5:** Stepwise regression coefficients and probability values for 4 steps of correlating 26 behaviours with the TBRWI.

Step	1	2	3	4
Constant	0.87	0.78	0.82	0.73
Inactivity	−0.00031	−0.00027	−0.00032	−0.00026
*p*	0.03	0.03	0.006	0.01
Startle		0.012	0.013	0.010
*p*		0.05	0.02	0.05
Box walking			−0.0029	−0.0035
*p*			0.03	0.01
Elimination				0.0028
*p*				0.08
S	0.122	0.105	0.086	0.074
R^2^	36.3	56.7	74.3	82.9

**Table 6:** Trainers’ (*n* = 58) demographics in Study 2.

Q1	Gender	Count	%
	Male	43	74.1
	Female	15	25.9
Q2	Age		
	<19	1	1.7
	19–24	2	3.5
	25–30	5	9.0
	31–40	6	10.3
	41–50	18	31.0
	51–61	26	45.0
Q3	Highest level of education		
	Primary school	3	5.2
	High school	27	46.6
	Technical and further education college	14	24.1
	University	14	24.1
	Other, please specify	0	0
Q4	Total Time of Involvement with TBRs		
	<1 month	2	3.5
	1–12 months	1	1.7
	13–48 months	1	1.7
	>48 months	54	93.1
Q5	Where did you mostly gain this experience?		
	Australia	53	91.4
	United Kingdom	0	0
	Japan	0	0
	France	1	1.7
	Ireland	0	0
	Germany	0	0
	Other, please specify	4	7.0

**Table 7:** Responses of trainers (*n* = 58) in the survey of welfare issues and levels for their horses.

Issue	Level	No. of Trainers	%
Q1. Horsemanship How experienced are your staff?	0. Failed to answer question	1	2.0
	1. All staff are experienced and well-trained, employing knowledge of equine behaviour in management and training	47	81.0
	2. Approximately half the staff are experienced and have the ability to evaluate health and welfare	7	12.0
	3. No staff are able to evaluate health and welfare and frequently resort to force	3	5.2
Q2. Health and Disease How well are your horses’ health and disease problems attended to?	1. Regular attention to health, appropriate use of analgesics, tranquilizers and parasitic control	55	95.0
	2. Some attention to health, occasional use of analgesics, tranquilizers and parasitic control medication	2	3.5
	3. Infrequent attention to health, analgesics, tranquilizers and parasitic control medication used only when absolutely necessary	1	2.0
Q3. Education of the Thoroughbred Racehorse To what level are your horses educated when sent to you for race training?	0. Unanswered	1	2.0
	1. Regular training from birth through to weaning sales preparation and transporting, riding, track work, barrier habituation, and racing	31	54.0
	2. Some handling as a foal, through to weaning sa2es preparation and transportation, riding, track work, barrier habituation and racing.	24	41.4
	3. No handling as a foal or weanling, little preparation for sales and transporting, riding and track work rushed with no habituation to barrier	2	3.5
Q4. Track Design and Surface What type of track do your horses race on?	1. Gradual turning cambered turf track	26	45.0
	2. Gradual turning cambered synthetic track	4	7.0
	3. Tight turning cambered turf track.	8	14.0
	4. Tight turning cambered synthetic	3	6.0
	5. Other, please describe	17	29.3
Q5. Ventilation How good is the ventilation for your horses?	1. Good ventilation: fans in every stable good ventilation in transport	29	50.0
	2. Some ventilation: fans at end of stable corridors some ventilation in transport	10	17.2
	3. Poor ventilation: stable walls of solid construction to at least 110 cm with wire mesh above; inadequate ventilation	2	3.5
	4. Other, please describe	17	29.3
Q6. Stabling How much space do your horses have?	1. Large stable (approximately 5 m × 5 m with ceiling height approximately 6m), with free use of attached yard.	21	36.2
	2. Small stable (approximately 3.6 m × 3.6 m with ceiling height approximately 4m) with free use of attached yard.	3	5.2
	3. Large stable (approximately 5 m × 5 m with ceiling height approximately 6m), with no use of attached yard.	14	24.1
	4. Small stable (approximately 3.6 m × 3.6 m with ceiling height approximately 4 m) with no use of attached yard.	8	14.0
	5. Other, please describe	12	21.0
Q7. Weaning Which weaning process was used for the majority of the horses that you train?	1. Removal of one mare at a time from a group of mares and foals in a paddock, until all mares are removed from group	8	14.0
	2. Two weanlings isolated together in a stable with visual and physical contact with neighbouring horses	13	22.4
	3. One weanling in a stable with no visual or physical contact with neighbouring horses.	3	5.2
	4. Unknown	30	52.0
	5. Other, please describe	4	7.0
Q8. Transport How skilled are your transport drivers?	1. Skilled driver, very experienced in loading and offloading horses	52	90.0
	2. Semi-skilled driver, some experience with loading and offloading horses	4	7.0
	3. Staff with limited experience in driving, loading and offloading horses	1	2.0
	4. Other, please describe	1	2.0
Q9. Nutrition Do you tailor your horses’ nutritional needs to their requirements (including giving green forage)?	1. Attention to age and training requirements of individual horses in order to balance fibre/grain intake with addition of proven supplement requirements and access to additional green forage	45	78.0
	2. Attention to age and training requirements of individual horses in order to balance fibre/grain intake with proven supplement requirements, infrequent access to additional green forage	10	17.2
	3. Standard nutritional program for all horses regardless of racing program, no additional green forage	1	2.0
	4. Other, please describe	2	3.5
Q10. Wastage What happens to your horses when retired from racing?	1. Horse retired from racing to a breeding farm	10	17.2
	2. Horse retired for equestrian sports	19	33.0
	3. Horse given away as race record was insufficient for breeding or temperament unsuitable for retraining in equestrian sports	8	14.0
	4. Horse sent to slaughterhouse, unsuitable for further use	1	2.0
	5. Unknown or other, please describe	20	34.5
Q11. Heat and Humidity Are your horses exposed to extreme climatic conditions?	1. Horses are regularly exposed to climatic variations; inadequate acclimatization following transport; poor stable design for temperature control	5	9.0
	2. Horses sometimes exposed to climatic variation; some acclimatization following transport; stable design allows for some temperature control	22	38.0
	3. Horses rarely exposed to climatic variations; good acclimatization following transport, stable design allows for good temperature control	29	50.0
	4. Other, please describe	2	3.4
Q12. Whips How often are your horses whipped in their races?	1. Whipping horses occasionally throughout the race	36	62.07
	2. Whipping horses regularly in the last 100 m of the race if they are tired	12	21.0
	3. No use of whip, jockeys ride with ‘hands and heels’	10	17.2
Q13. Environment What environment are the horses kept in?	1. Wood shavings. Stable/yard design allows only visual contact with other horses	27	47.0
	2. Wood shavings. Stable/yard design allows physical and visual contact with other horses	17	29.3
	3. Straw bedding. Stable/yard design allows physical and visual contact with other horses	2	3.5
	4. Straw bedding. Stable/yard design allows only visual contact with other horses	1	2.0
	5. Other, please specify	11	19.0
Q14. Gear Do you use blinkers and tongue tie routinely?	1. No blinkers or tongue tie	14	24.1
	2. Use of blinkers, but no tongue tie	9	15.5
	3. Use of tongue tie and blinkers	34	59.0
	4. Use of tongue tie but no blinkers	1	2.0

**Table 8:** Composition of TBRWI from utility values obtained from issues, levels and their level of importance values from the Thoroughbred racehorse husbandry questionnaire and the trainer survey.

Issues		Levels (Abbreviated from Table 7)	Utility Values	Contribution to TBRWI
Horsemanship	1	All staff are experienced	62.29	8.80
	2	50% of staff are experienced	−3.89	3.97
	3	None of the staff are experienced	−58.40	0
Health and disease	1	Regular attention to health	62.35	8.50
	2	Some attention to health	−14.68	2.55
	3	Infrequent attention to health	−47.66	0
Education of horse	1	Regular training	52.80	8.50
	2	Some handling	7.38	5.08
	3	No handling	−60.18	0
Ventilation	1	Good ventilation	48.72	8.00
	2	Some ventilation	6.12	4.71
	3	Poor ventilation	−54.84	0
Track design and surface	1	Gradual-turning turf track	51.01	7.60
	2	Gradual-turning synthetic track	27.03	7.01
	3	Tight-turning turf track	−33.12	1.15
	4	Tight-turning synthetic track	−44.91	0
Weaning	1	Two weanlings together in stable	26.65	7.55
	2	Removal of one mare at a time	24.29	7.32
	3	One weanling/stable	−50.94	0
Nutrition	1	Attention to individual horse, access to forage	47.81	7.45
	2	Attention to individual horse, infrequent access to forage	0.85	3.82
	3	Standard nutritional program for all horses, no forage	−48.66	0
Stabling	1	Large stable with yard	37.72	7.35
	2	Small stable with yard	14.26	5.23
	3	Large stable without yard	−8.39	3.19
	4	Small stable without yard	−43.59	0
Transport	1	Skilled driver	51.43	7.25
	2	Semi-skilled driver	−7.98	3.43
	3	Staff with limited experience	−43.45	0
Wastage	1	Horses retired to equestrian sports	30.44	7.15
	2	Horses retired to a breeding farm	22.85	6.34
	3	Horse given away	−16.37	2.18
	4	Horse sent to a slaughterhouse	−36.92	0
Heat and humidity	1	Rarely exposed to climatic variation	31.25	6.85
	2	Sometimes exposed to climatic variation	12.37	5.10
	3	Regularly exposed to climatic variations	−43.63	0
Whips	1	Whipping occasionally throughout race	23.71	6.55
	2	No use of whip	10.91	5.11
	3	Whipping in the last 100 m	−34.61	0
Environment	1	Wood shavings, only visual contact	3.44	6.00
	2	Wood shavings, physical and visual contact	0.10	3.72
	3	Straw bedding, physical and visual contact	−1.45	1.09
	4	Straw bedding, only visual contact	−2.09	0
Gear	1	No blinkers or tongue tie	14.74	2.80
	2	Blinkers, no tongue tie	1.57	1.23
	3	Tongue tie, no blinkers	−8.76	0

**Figure A1:**
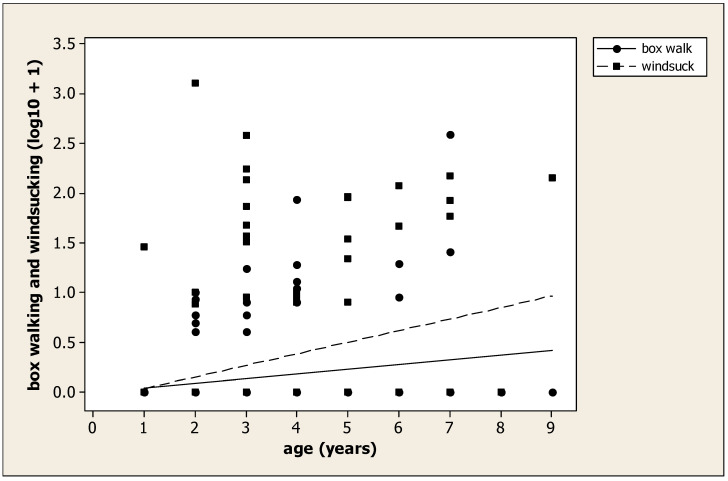
Changes in box walking (log_10_ % time) and windsucking (log_10_ % time) with age in years. Linear regression lines indicated.

## Data Availability

Data is available from the authors on request.

## Abbreviations

Elim	Elimination
Aggress	Aggression
Boxwalk	Boxwalking
Windsuck	Windsucking
Chewob	Chewing on objects
Shamchew	Sham chewing
Outofsight	Out of sight
St. sleep	Standing sleep
St. inactive	Standing inactive
St. resting	Standing resting
Headtoss	Head toss

## Appendix A

**Table A1:** Description of the individual stables of the training stables (*n* = 13) in the survey of TBR behaviour.

Stable	Length (m)	Width (m)	Height (m)	Notes
**1**	5.0	5.0	6.4	Barn design, corridor down the middle. Attached yard not in use. No fans
**2**	5.0	5.0	6.4	Barn design. Corridor down the middle. Free use of attached yard. No fans
**3**	6.0	2.2	3.7	Yard with covered shelter at far end of yard. Physical and visual contact. No fans
**4**	3.7	3.6	2.6	Side by side design. No grass. No yard. Visual contact only. No fans
**5**	6.4	3.7	2.2	Yard with covered shelter at far end. Physical and visual contact. No fans
**6**	3.8	3.7	3.4	Barn design. No yards. Fans in corridor. Physical and visual contact
**7**	3.7	3.6	3.5	Side by side design, grassed area. No physical or visual contact.
**8**	3.8	3.7	3.4	Barn design. No yards. Fans in corridor. Physical and visual contact
**9**	5.9	3.8	3.5	Side by side design. Yard partly covered. No fans. Physical and visual contact between horses.
**10**	5.4	4.7	4.0	Open barn cluster design. 3 yards not covered, shaded by trees. No fans. Visual contact. Not all stables had physical contact.
**11**	4.4	4.3	4.2	Barn design. Some yards. External air vent to ceiling. Physical and visual contact
**12**	3.7	3.3	3.0	Side by side design. Some yards. Ceiling fans. Physical and visual contact
**13**	5.1	4.9	4.2	Open barn, Heavy wire mesh, self-ventilating. Physical and visual contact. Side by side and back to back design.

## Appendix B. Questionnaire Sent to Trainers to Indicate Welfare Issues and Levels within the Thoroughbred Horse Racing Industry

**SECTION 1—DEMOGRAPHICS**

Q1 Gender:

Please select from the following.

□ Male
□ Female

Q2 Please select your age group:

□ Under 19 years
□ 19–24
□ 25–30
□ 31–40
□ 41–50
□ 51–60
□ 61+

Q3 Please select your highest achieved level of education:

□ Primary School
□ High School
□ Technical and further education college
□ University
□ Other, please specify_____________________________________

Q4 What is your total time of involvement with Thoroughbred racehorses?

□ Less than 1 month
□ 1–12 months
□ 13–48 months
□ Over 48 months

Q5 Where did you mostly gain this experience?

□ Australia
□ United Kingdom
□ Japan
□ France
□ Ireland
□ Germany
□ Other, please specify_____________________________________

**SECTION 2—WELFARE AND MANAGEMENT**

Q1 Horsemanship

How experienced are your staff?

□ All staff are experienced and well trained, employing knowledge of equine behaviour in management and training.
□ Approximately half of the staff are experienced and have the ability to evaluate health and welfare.
□ None of the staff have the ability to evaluate health and welfare and frequently resort to force.

Q2 Health and Disease

□ How well are your horses’ health and disease problems attended to?
□ Regular attention to health. Appropriate use of analgesics, tranquilizers and parasitic control.
□ Some attention to health. Occasional use of analgesics, tranquilizers and parasitic control medication.
□ Infrequent attention to health. Analgesics, tranquilizers and parasitic control medication used only when absolutely necessary.

Q3 Education of Horses

To what level are your horses educated when sent to you for race training?

□ Regular training from birth through to weaning, sales preparation and transporting, riding, track work, barrier habituation, and racing.
□ Some handling as a foal, through to weaning, sales preparation and transportation, riding, track work, barrier habituation and racing.
□ No handling as a foal or weanling. Little preparation for sales and transporting. Riding and track work rushed with no habituation to barrier

Q4 Track Design and Surface

What type of track do your horses race on?

□ Gradual turning cambered turf track.
□ Gradual turning cambered synthetic track.
□ Tight turning cambered turf track.
□ Tight turning cambered synthetic track.
□ Other, please describe_____________________________________

Q5 Ventilation

How good is the ventilation for your horses?

□ Good ventilation: fans in every stable; good ventilation in transport.
□ Some ventilation: fans at end of stable corridors; some ventilation in transport.
□ Poor ventilation: stable walls of solid construction to at least 110cm with wire mesh above; inadequate ventilation in transport.
□ Other, please describe______________________________________________

Q6 Stabling

How much space do your horses have?

□ Large stable (approximately 5 m × 5 m with ceiling height approximately 6 m), with free use of attached yard.
□ Small stable (approximately 3.6 m × 3.6 m with ceiling height approximately 4 m) with free use of attached yard.
□ Large stable (approximately 5 m × 5 m with ceiling height approximately 6 m), with no use of attached yard.
□ Small stable (approximately 3.6 m × 3.6 m with ceiling height approximately 4 m) with no use of attached yard.
□ Other, please describe___________________________________________________

Q7 Weaning

Which weaning process was used for the majority of the horses that you train?

□ Removal of one mare at a time from a group of mares and foals in a paddock, until all mares are removed from group.
□ Two weanlings isolated together in a stable which allows visual and physical contact with neighbouring horses.
□ One weanling in a stable which does not allow visual or physical contact with neighbouring horses.
□ Unknown
□ Other, please describe_________________________________________________

Q8 Transport

How skilled are your transport drivers?

□ Skilled driver, very experienced in loading and offloading horses.
□ Semi-skilled driver, some experience of loading and offloading horses.
□ Staff with limited experience in driving, loading and offloading horses.
□ Other, please describe_________________________________________________

Q9 Nutrition

Do you tailor your horses’ nutritional needs to their requirements, including giving green forage?

□ Attention to age and training requirements of individual horses in order to balance fibre/grain intake with addition of proven supplement requirements and access to additional green forage.
□ Attention to age and training requirements of individual horses in order to balance fibre/grain intake with proven supplement requirements, infrequent access to additional green forage.
□ Standard nutritional program for all horses regardless of racing programme, no additional green forage.
□ Other, please describe__________________________________________________

Q10 Wastage

What happens to your horses when retired from racing?

□ Horse retired from racing to a breeding farm.
□ Horse retired for equestrian sports.
□ Horse given away as race record was insufficient for breeding or temperament unsuitable for retraining in equestrian sports.
□ Horse sent to slaughterhouse, unsuitable for further use.
□ Unknown or other, please describe________________________________________

Q11 Heat and Humidity

Are your horses exposed to extreme climatic conditions?

□ Horses are regularly exposed to climatic variations; inadequate acclimatisation following transport; poor stable design for temperature control.
□ Horses sometimes exposed to climatic variation; some acclimatisation following transport; stable design allows for some temperature control.
□ Horses rarely exposed to climatic variations; good acclimatisation following transport; stable design allows for good temperature control.
□ Other, please describe__________________________________________________

Q12 Whips

How often are your horses whipped in their races?

□ Whipping horses occasionally throughout the race.
□ Whipping horses regularly in the last 100 m of the race if they are tired.
□ No use of whip, jockeys ride with hands and heels.

Q13 Environment

What is the environment in which the horses are kept?

□ Use of wood shavings. Stable/yard design allows only visual contact with other horses.
□ Use of wood shavings. Stable/yard design allows physical and visual contact with other horses.
□ Use of straw bedding. Stable/yard design allows physical and visual contact with other horses.
□ Use of straw bedding. Stable/yard design allows only visual contact with other horses.
□ Other, please specify___________________________________________________

Q14 Gear

Do you use blinkers and tongue tie routinely?

□ No blinkers or tongue tie.
□ Use of blinkers, but no tongue tie
□ Use of tongue tie and blinkers.
□ Use of tongue tie but no blinkers.

**END OF SURVEY**

## Appendix C

**Table A2:** Pearson correlation coefficients of transformed behaviour values (log_10_ behaviour + 1, top value) and probability that the correlation is significant (bottom value).

	Drink	Feeding	Elim	Lying	St. Resting	
Feeding	0.40					
	<0.001					
Elim	−0.02	−0.15				
	0.84	0.10				
Lying	−0.04	0.08	0.00			
	0.63	0.34	0.98			
Standing resting	0.24	−0.05	0.08	−0.15		
	0.01	0.61	0.33	0.08		
Standing inactive	0.01	−0.01	0.06	0.11	0.23	
	0.87	0.94	0.52	0.20	0.01	
Standing sleeping	−0.07	−0.01	0.11	0.17	0.23	
	0.40	0.90	0.23	0.06	0.01	
Rubbing	−0.01	−0.14	0.09	−0.01	0.17	
	0.87	0.10	0.31	0.88	0.06	
Aggress	−0.03	−0.01	0.24	−0.09	0.03	
	0.75	0.92	<0.001	0.30	0.72	
Vocal	−0.08	0.00	0.11	0.06	0.09	
	0.33	0.98	0.21	0.53	0.30	
Sniffing	0.10	0.16	0.25	−0.11	0.20	
	0.27	0.07	<0.001	0.20	0.02	
Boxwalk	−0.20	−0.22	0.13	0.07	−0.10	
	0.02	0.01	0.15	0.44	0.23	
Walk	−0.25	−0.24	0.15	0.06	−0.11	
	<0.001	0.01	0.08	0.51	0.22	
Play	0.05	0.10	0.11	0.07	0.07	
	0.54	0.25	0.20	0.41	0.44	
Windsuck	0.01	−0.24	0.02	0.02	0.14	
	0.95	0.01	0.84	0.82	0.11	
Chewob	0.07	−0.20	0.07	0.12	0.23	
	0.46	0.02	0.42	0.19	0.01	
Shamchew	0.13	0.08	0.14	0.18	0.02	
	0.13	0.34	0.11	0.04	0.85	
Licking	−0.03	−0.01	0.13	0.02	−0.13	
	0.70	0.93	0.15	0.80	0.15	
Yawn	−0.03	0.02	0.07	−0.08	−0.02	
	0.78	0.79	0.43	0.38	0.78	
Weave	−0.33	−0.45	0.26	−0.17	−0.20	
	<0.001	<0.001	<0.001	0.05	0.02	
Doorbang	0.00	−0.10	0.05	−0.06	0.05	
	0.97	0.24	0.58	0.47	0.54	
Headtoss	−0.27	−0.11	0.02	0.05	0.05	
	<0.001	0.22	0.84	0.58	0.54	
Paw	−0.00	−0.08	0.11	−0.12	0.11	
	1.00	0.37	0.23	0.17	0.22	
Look	−0.07	0.02	0.09	0.03	−0.02	
	0.42	0.85	0.32	0.73	0.85	
Startle	−0.02	−0.05	0.04	0.02	0.11	
	0.85	0.60	0.65	0.79	0.21	
Outofsight	0.18	0.19	−0.18	0.01	−0.26	
	0.04	0.03	0.04	0.88	<0.001	
	**St. inactive**	**St. sleep**	**Rubbing**	**Aggress**	**Vocal**	
St. sleep	0.21					
	0.02					
Rubbing	0.02	−0.10				
	0.80	0.28				
Aggress	0.11	0.08	0.22			
	0.20	0.38	0.01			
Vocal	−0.01	0.25	0.07	−0.01		
	0.91	<0.001	0.40	0.91		
Sniffing	0.05	0.08	0.26	0.27	0.08	
	0.59	0.35	<0.001	<0.001	0.35	
Boxwalk	0.03	0.07	0.06	0.01	0.25	
	0.78	0.46	0.51	0.91	0.00	
Walk	−0.04	0.16	0.02	−0.05	0.14	
	0.62	0.07	0.79	0.55	0.10	
Play	0.11	0.01	0.26	0.16	0.18	
	0.19	0.91	<0.001	0.07	0.04	
Windsuck	0.17	0.17	−0.07	−0.04	0.01	
	0.05	0.05	0.43	0.63	0.91	
Chewob	0.17	0.20	0.15	−0.04	0.02	
	0.05	0.02	0.09	0.62	0.81	
Sham chew	0.09	0.11	0.087	0.12	0.01	
	0.29	0.20	0.32	0.18	0.88	
Licking	0.04	0.15	0.14	0.10	−0.03	
	0.68	0.08	0.10	0.25	0.73	
Yawn	−0.12	0.02	0.18	−0.01	−0.07	
	0.22	0.79	0.03	0.96	0.44	
Weave	−0.08	−0.12	0.00	0.10	−0.04	
	0.36	0.18	0.98	0.24	0.67	
Doorbang	0.09	0.05	−0.01	−0.03	−0.03	
	0.33	0.57	0.92	0.71	0.77	
Headtoss	−0.01	0.16	0.15	0.13	0.25	
	0.91	0.06	0.08	0.14	0.00	
Paw	−0.19	0.00	0.12	0.12	0.03	
	0.03	0.99	0.18	0.17	0.77	
Look	0.02	0.16	0.18	0.12	0.15	
	0.84	0.07	0.04	0.17	0.09	
Startle	0.01	0.12	0.04	0.13	0.09	
	0.88	0.17	0.65	0.15	0.32	
Outofsight	−0.29	−0.14	−0.21	−0.22	−0.13	
	<0.001	0.11	0.01	0.01	0.14	
	**Sniffing**	**Boxwalk**	**Walk**	**Play**	**Windsuck**	
Boxwalk	−0.02					
	0.78					
Walk	−0.07	0.06				
	0.42	0.50				
Play	0.16	0.20	0.04			
	0.06	0.02	0.63			
Windsuck	0.01	0.06	−0.09	−0.09		
	0.91	0.47	0.32	0.33		
Chewob	−0.09	−0.12	0.07	0.07	0.26	
	0.29	0.18	0.43	0.45	<0.001	
Sham chew	0.06	0.09	0.12	0.23	−0.10	
	0.51	0.30	0.17	0.01	0.26	
Licking	0.06	0.12	0.14	0.05	−0.04	
	0.49	0.16	0.10	0.56	0.62	
Yawn	0.16	−0.06	0.07	0.00	−0.09	
	0.07	0.51	0.41	0.97	0.29	
Weave	−0.05	0.16	0.25	0.05	0.09	
	0.61	0.07	<0.001	0.53	0.31	
Doorbang	−0.01	0.08	0.04	0.12	−0.08	
	0.88	0.35	0.65	0.17	0.39	
Headtoss	0.07	0.16	0.07	0.08	−0.11	
	0.43	0.07	0.42	0.34	0.22	
Paw	0.19	−0.01	0.10	0.19	0.07	
	0.03	0.95	0.26	0.03	0.40	
Look	0.16	0.18	0.13	0.09	0.10	
	0.06	0.04	0.14	0.29	0.25	
Startle	0.04	0.08	0.04	−0.01	−0.02	
	0.67	0.36	0.65	0.94	0.83	
Outofsight	−0.21	−0.07	0.06	−0.14	−0.20	
	0.02	0.41	0.51	0.10	0.03	
	**Chewob**	**Sham chew**	**Licking**	**Yawn**	**Weave**	
Sham chew	0.12					
	0.15					
Licking	−0.01	0.08				
	0.96	0.39				
Yawn	−0.01	0.13	0.16			
	0.88	0.15	0.07			
Weave	−0.03	−0.09	0.27	−0.11		
	0.70	0.30	<0.001	0.21		
Doorbang	−0.11	0.03	0.14	0.06	0.07	
	0.23	0.72	0.11	0.47	0.42	
Headtoss	0.00	0.04	0.05	0.10	−0.00	
	0.97	0.68	0.56	0.23	0.96	
Paw	0.08	0.08	0.06	0.05	0.09	
	0.39	0.37	0.53	0.60	0.30	
Look	0.12	0.06	0.12	0.08	−0.15	
	0.17	0.50	0.18	0.34	0.09	
Startle	0.08	0.05	−0.04	0.15	−0.00	
	0.35	0.57	0.64	0.09	0.96	
Outofsight	−0.15	0.03	−0.09	0.10	−0.09	
	0.09	0.77	0.32	0.25	0.31	
	**Doorbang**	**Headtoss**	**Paw**	**Look**	**Startle**	
Headtoss	−0.10					
	0.24					
Paw	0.05	0.05				
	0.56	0.54				
Look	0.10	0.14	0.06			
	0.28	0.12	0.48			
Startle	0.16	0.21	−0.06	0.16		
	0.06	0.02	0.52	0.07		
Outofsight	−0.02	−0.20	−0.02	−0.23	0.11	
	0.84	0.03	0.82	0.01	0.2	

## Appendix E

**Table A3:** Stepwise regression coefficients and probability values for the two steps (addition of feeding first and then drinking), when regressing mean behaviour values with mean values for the welfare issues in the TBRWI.

Step	1	2
Constant	0.27	0.23
Feeding	0.00003	0.00006
*Probability*	0.05	0.003
Drinking		−0.00028
*Probability*	0.164	0.02
S	0.164	0.127
R^2^	30.5	62.3

## References

[1] Waran N., McGreevy P.D., Casey R.A., Waran N. (2007). Training methods and horse welfare. The Welfare of Horses.

[2] Boden L. (2008). Risk Factors Associated with Racetrack Casualties in Thoroughbreds: Victoria, Australia 1989–2005.

[3] Australian Racing Board Ltd (2014). A Guide to the Racing Industry in Australia.

[4] Rural Industries Research and Development Council (2006). Horse R&D Plan 2006 to 2011 (Document Number 06/114).

[5] Sainsbury D.W.B., Hickman J. (1987). Housing the horse. Horse Management.

[6] Pritchard J.C., Lindberg A.C., Main D.C., Whay H.R. (2005). Assessment of the welfare of working horses, mules and donkeys, using health and behaviour parameters. Prev. Vet. Med..

[7] Petersen S., Tolle K.H., Blobel K., Grabner A., Krieter J. (2006). Evaluation of horse keeping in Schleswig-Holstein. Zuchtungskunde.

[8] Christie J.L., Hewson C.J., Riley C.B., McNiven M.A., Dohoo I.R., Bate L.A. (2006). Management factors affecting stereotypies and body condition score in nonracing horses in Prince Edward Island. Can. Vet. J..

[9] Collins J.A., Hanlon A., More S.J., Wall P.G., Kennedy J., Duggan V. (2010). Evaluation of current equine welfare issues in Ireland: Causes, desirability, feasibility and means of raising standards. Equine Vet. J..

[10] Tadich T., Weber C., Nicol C.J. (2012). Prevalence and Factors Associated with Abnormal Behaviors in Chilean Racehorses: A Direct Observational Study. J. Equine Vet. Sci..

[11] (2013). Racing Queensland Magazine.

[12] Cooper J.J., Mason G.J. (1998). The identification of abnormal behaviour and behavioural problems in stabled horses and their relationship to horse welfare: A comparative view. Equine Vet. J. Suppl..

[13] Mills D.S., Clarke A., Waran N. (2007). Housing, Management and Welfare. The Welfare of Horses.

[14] Altmann J. (1974). Observational study of behaviour sampling methods. Behaviour.

[15] McGreevy P.D., French N.P., Nicol C.J. (1995). The prevalence of abnormal behaviors in dressage, eventing and endurance horses in relation to stabling. Vet. Rec..

[16] Cooper J., McGreevy P., Waran N. (2007). Stereotypical behaviour in the stabled horse: Causes, effects and prevention without compromising horse welfare. The Welfare of Horses.

[17] Ransom J.I., Cade B. (2009). Quantifying Equid Behaviour—A Research Ethogram for Free-Roaming Feral Horses (Document Number A9).

[18] McDonnell S.M. (2003). The Equid Ethogram: A Practical Field Guide to Horse Behavior.

[19] Houpt K.A. (2011). Domestic Animal Behaviour for Veterinarians and Animal Scientists.

[20] Petherick C., Mills D.S. (2010). Sleep. Encyclopedia of Applied Animal Behaviour and Welfare.

[21] Racehorse Trainers from Study 2 (2022). Personal Communication.

[22] Dallaire A. (1986). Rest behavior. Vet. Clin. North Am.-Equine Pract..

[23] Heleski C.R., Shelle A.C., Nielsen B.D., Zanella A.J. (2002). Influence of housing on weanling horse behavior and subsequent welfare. Appl. Anim. Behav. Sci..

[24] Dodman N.H., Normile J.A., Cottam N., Guzman M., Shuster L. (2005). Prevalence of compulsive behaviors in formerly feral horses. Int. J. Appl. Res. Vet Erinary Med..

[25] Evans D.L., Waran N. (2007). Welfare of the Racehorse during Exercise Training and Racing. The Welfare of Horses.

[26] Hall C., Goodwin D., Heleski C., Randle H., Waran N. (2008). Is there evidence of learned helplessness in horses?. J. Appl. Anim. Welf. Sci..

[27] Pellis S.M., Pellis V.C., Mills D.S. (2010). Sniffing. Encyclopedia of Applied Animal Behaviour and Welfare.

[28] McGreevy P. (2004). Equine Behavior: A Guide for Veterinarians and Equine Scientists.

[29] McDonnell S.M., Haviland J.C.S. (1995). Agnostic ethogram of the equid bachelor band. Appl. Anim. Behav. Sci..

[30] Goodwin D., Waran N. (2007). Horse Behaviour: Evolution, Domestication and Feralisation. The Welfare of Horses.

[31] Rees L. (1993). The Horse’s Mind.

[32] Weary D., Mills D.S. (2010). Vocalisation. Encyclopedia of Applied Animal Behaviour and Welfare.

[33] Flower F., Mills D.S. (2010). Locomotion. Encyclopedia of Applied Animal Behaviour and Welfare.

[34] Mason G.J. (1991). Stereotypies: A critical review. Anim. Behav..

[35] Willard J.G., Willard J.C., Wolfram S.A., Baker J.P. (1977). Effect of diet on cecal pH and feeding behavior of horses. J. Anim. Sci..

[36] Kiley-Worthington M. (1983). Stereotypies in the horse. Equine Pract..

[37] Mills D.S., Nankervis K.J. (1999). Equine Behaviour: Principles and Practice.

[38] Kiley-Worthington M. (1987). The Behaviour of Horses.

[39] Cooper J.J., McDonald L., Mills D.S. (2000). The effect of increasing visual horizons on stereotypic weaving: Implications for the social housing of stabled horses. Appl. Anim. Behav. Sci..

[40] Mactaggart G., Waran N., Phillips C.J.C. (2021). Identification of thoroughbred racehorse welfare issues by industry stakeholders. Animals.

[41] Barter C., Renold E. (2000). ‘I wanna tell you a story’: Exploring the application of vignettes in qualitative research with children and young people. Int. J. Soc. Res. Methodol..

[42] Wilks T. (2004). The use of vignettes in qualitative research into social work values. Qual. Soc. Work.

[43] Sawtooth Software. www.sawtoothsoftware.com.

[44] Minitab, LLC. (2021). Getting Started with Minitab. www.minitab.com.

[45] Casey R.A., Waran N. (2007). Clinical Problems in Performance Horses. The Welfare of Horses.

[46] Marsden M.D. (1995). An investigation of the heredity of susceptibility of stereotypic behaviour pattern-stable vices in the horse. Equine Vet. J..

[47] Boy V., Duncan P. (1979). Time-budgets of camargue horses. 1. Developmental-changes in the time-budgets of foals. Behaviour.

[48] Duncan P. (1985). Time-budgets of Camargue horses. 3. Environmental-influences. Behaviour.

[49] Boyd L.E., Carbonaro D.A., Houpt K.A. (1988). The 24-hour time budget of Przewalski horses. Appl. Anim. Behav. Sci..

[50] Golland L.C., Evans D.L., Stone G.M., Tyler-McGowan C.M., Hodgson D.R., Rose R.J. (1999). Plasma cortisol and beta-endorphin concentrations in trained and over-trained standardbred racehorses. Pflug. Arch..

[51] Taylor K., Mills D.S. (2010). Applied Ethology Disorders of Behaviour. Encyclopedia of Applied Animal Behaviour and Welfare.

[52] Ecke P., Hodgson D.R. (1996). Survey of the incidence of acute colitis in adult horses in Australia. Aust. Equine Vet..

[53] Sjaastad O., Hove K., Sand O. (2003). Physiology of Domestic Animals.

[54] Lester G.D., Robertson I., Secombe C. (2008). Risk Factors for Gastric Ulceration in Thoroughbred Racehorses.

[55] Aleman M., Thomas B., Magdesian G., Ferraro G., Sonder C., Whitcomb M.B., Kozikowski-Nicholas T. (2013). The Equine Heart: Power Plant Unequalled.

[56] Gordon J. (2001). The Horse Industry: Contributing to the Australian Economy (Document Number 01/083).

[57] Hayek A. (2004). Epidemiology of Horses Leaving the Racing and Breeding Industries. Bachelor’s Thesis.

[58] Bartussek H. (1995). Animal needs Index for Cattle (Document Number TGI35L).

[59] Johnsen P.F., Johannesson T., Sandøe P. (2001). Assessment of Farm Animal Welfare at Herd Level: Many Goals, Many Methods. Acta Agric. Scand. Sect. A Anim. Sci..

[60] Dawkins M.S., Baxter S.H., Baxter M.R., MacCormack J.A.D. (1983). The Current Status of Preference Tests in the Assessment of Animal Welfare. Farm Animal Housing and Welfare.

[61] Leighton-Hardman A.C. (1984). Equine Nutrition.

[62] Kohnke J., Kelleher F., Trevor-Jones P. (1999). Feeding Horses in Australia: A Guide for Horse Owners and Managers (Document Number UWS-13A).

[63] Davidson N., Harris P., Waran N. (2007). Nutrition and Welfare. The Welfare of Horses.

[64] Racklyeft D.J., Love D.N. (1990). Influence of head posture on the respiratory-tract of healthy horses. Aust. Vet. J..

[65] Cullinane B., Sprayberry K.A., Robinson N.E. (1987). Viral respiratory disease. Robinson’s Current Therapy in Equine Medicine.

[66] Collins M.N., Jousan F.D., Chen S.C., Friend T.H. (1999). Effects of density on displacement, falls, injuries, and orientation during horse transportation. J. Anim. Sci..

[67] Rollin B.E. (2000). Equine welfare and emerging social ethics. J. Am. Vet. Med. Assoc..

[68] Waran N., Leadon D., Friend T., Waran N. (2007). The Effects of Transportation on the Welfare of Horses. The Welfare of Horses.

[69] Cabib S., Mason G., Rushen J. (2006). The neurobiology of stereotypy II: The role of stress. Stereotypic Animal Behavior: Fundamentals and Applications in Welfare.

[70] Dawkins M.S. (1980). Animal Suffering: The Science of Animal Welfare.

[71] Cooper J.J., Albentosa M.J. (2005). Behavioural adaptation in the domestic horse: Potential role of apparently abnormal responses including stereotypic behaviour. Livest. Prod. Sci..

[72] McLean A., McLean M. (2008). Academic Horse Training.

[73] Moyer W., Spencer P.A., Kallish M. (1991). Relative incidence of dorsal metacarpal disease in young thoroughbred racehorses training on 2 different surfaces. Equine Vet. J..

[74] Bailey C.J. (1998). Wastage in the Australian Thoroughbred Racing Industry.

[75] Whitton C.R., Holmes J., Mirams M., Mackie E. (2013). Bone Repair in Thoroughbred Racehorses the Effect of Training and Rest.

[76] Lees P., Higgins A.J. (1985). Clinical pharmacology and therapeutic uses of non-steroidal anti-inflammatory drugs in the horse. Equine Vet. Ournal.

[77] Clarke A.F., Hickman J. (1987). Stable environment in relation to the control of respiratory diseases. Horse Management.

[78] Van Erck-Westergren E., Franklin S.H., Bayly W.M. (2013). Respiratory diseases and their effects on respiratory function and exercise capacity. Equine Vet. J..

[79] Nicol C. (1999). Understanding equine stereotypies. Equine Vet. J. Suppl..

[80] Nicol C., Harris P.A., Gomarsall G.M., Davidson H.P.B., Green R.E. (1999). Stereotypies and Their Relation to Management. Proceedings of the 38th British Equine Veterinary Association Congress.

[81] Houpt K.A., Mills D., McDonnell S. (2005). Maintenance behaviors. The Domestic Horse: The Evolution, Development and Management of its Behaviour.

[82] Equestrian Australia (2018). Hot Weather Policy. https://www.equestrian.org.au/sites/default/files/EA_Hot_Weather_Policy_13042018.pdf.

[83] AHWC (2011). Australian Horse Welfare Protocol. Geelong Australia. www.ashs.com.au/media/1802/horse-welfare-protocol-policy.pdf.

[84] Queensland Government (2001). Animal Care and Protection Act (Section 17).

[85] Waters A.J., Nicol C.J., French N.P. (2002). Factors influencing the development of stereotypic and redirected behaviours in young horses: Findings of a four year prospective epidemiological study. Equine Vet. J..

[86] Holland J., Kronfeld D.S., Hoffman R., Greiwe-Crandell K.M., Boyd T., Cooper W.L., Harris P.A. (1996). Weanling stress is affected by nutrition and weanling methods. Pferdeheilkunde.

[87] Geelen R. (2013). Welfare of the Retired Thoroughbred, Facts vs Fiction. Breeding and Racing.

[88] Bailey C.J. (1998). Identification and Characterization of Causes of Wastage in the Australian Thoroughbred Racing Industry.

[89] Odberg F.O., Bouissou M.F. (1999). The development of equestrianism from the baroque period to the present day and its consequences for the welfare of horses. Equine Vet. J. Suppl..

[90] Holmes J.M., Mirams M., Mackie E.J., Whitton R.C. (2014). Thoroughbred horses in race training have lower levels of subchondral bone remodelling in highly loaded regions of the distal metacarpus compared to horses resting from training. Vet. J..

[91] McGreevy P.D. (1996). Paul McGreevy on Horse Behavior. Interviewed by RIRDC In RIRDC Equine Research News. http://www.equusite.com/articles/behavior/behaviorDrMcGreevy.shtml.

[92] Collins J.A., Hanlon A., More S.J., Wall P.G., Duggan V. (2009). Policy Delphi with vignette methodology as a tool to evaluate the perception of equine welfare. Vet. J..

[93] More S.J. (1999). A longitudinal study of racing thoroughbreds: Performance during the first years of racing. Aust. Vet. J..

[94] Ransom J.I. (2012). Population Ecology of Feral Horses in an Area of Fertility Control Management.

[95] Tyler S.J. (1972). The behaviour and social organisation of New Forest Ponies. Anim. Behav. Monogr..

[96] Ransom J.I., Wann M.E., Wilson J.T., Rowell J.E. (2007). America’s Wild Horses and Burros. Research for Management.

[97] Csurhes S., Paroz G., Markula A. (2009). Pest Animal Risk Assessment: Feral Horse Equus Caballus. Queensland Primary Industries and Fisheries. www.daf.qld.gov.au/__data/assets/pdf_file/0004/51961/IPA-Feral-Horses-Risk-Assessment.pdf.

[98] Hilmo N. (2013). Natural Feeding Strategies for Sport Horses A “Contradiction in Terms”?.

[99] Nicol C.J., Davidson H.P., Harris P.A., Waters A.J., Wilson A.D. (2002). Study of crib-biting and gastric inflammation and ulceration in young horses. Vet. Rec..

[100] Whip Use on Racehorses Questioned by Study Which Find They’re Thin-Skinned, Feel Pain. Sydney, New South Wales, Australian Broadcasting Corporation. 24 March 2015. https://www.abc.net.au/news/2015-03-24/whip-use-racehorses-questioned-study/6345296.

[101] Stewart J. (2012). Study Puts Horse Whipping under Scrutiny. Lateline.

[102] Butler R.K., Finn D.P. (2009). Stress-induced analgesia. Prog. Neurobiol..

[103] Wagner A.E. (2010). Effects of stress on pain in horses and incorporating pain scales for equine practice. Vet. Clin. North Am.-Equine Pract..

[104] Contino E.K., Khursheed R.M., Sprayberry K., Robinson N.E. (2015). Recognition of Pain. Robinson’s CurrentTherapy in Equine Medicine.

[105] Newby J. (2015). Horse Whip. Catalyst.

[106] McGreevy P.D., Corken R.A., Salvin H., Black C.M. (2012). Whip Use by Jockeys in a Sample of Australian Thoroughbred Races-An Observational Study. PLoS ONE.

[107] Payne C. (2013). Cox Plate Winning Jockey Fine for Excessive Whip Use. Racenet.com.au (26/08/2013). www.racenet.com.au/news/94584/Cox-Plate-winning-jockey-fined-for-excessive-whip-use.

[108] Exelby N. (2015). Jockeys Whipping Hand that Feeds as Luke Tarrant and Tim Bell Cop Suspensions on Magic Millions Day. The Courier Mail Newspaper.

[109] McGauran P. (2015). Horse Whip.

[110] McGreevy P.D., McLean A.N. (2018). Apparatus. Equitation Science.

[111] Mills D.S. (1998). Applying learning theory to the management of the horse: The difference between getting it right and getting it wrong. Equine Vet. J. Suppl..

[112] McGreevy P.D., McLean A.N. (2010). Equitation Science.

[113] Feist J.D., McCullough D.R. (1976). Behavior patterns and communication in feral horses. Z. Für Tierpsychol..

[114] Crowell-Davis S.L. (1993). Social behaviour of the horse and its consequences for domestic management. Equine Vet. Educ..

[115] Mills D., Eckley S., Cooper J.J. (2000). Thoroughbred bedding preferences, associated behaviour differences and their implications for equine welfare. Anim. Sci..

[116] Clarke A.F. (1987). Chronic pulmonary disease—A multi-faceted disease complex in the horse. Ir. Vet. J..

[117] Beyer S. (1998). Konstruktion und Überprüfung eines Bewertungskonzeptes für Pferdehaltende Betriebe unter dem Aspekt der Tiergerechtheit. Ph.D. Thesis.

[118] Kohnke J.R. (2005). Talking horses: Racing edition. Issue 6. http://www.kohnkesown.com.

[119] Houpt K.A., Hintz H.F., Butler W.R. (1984). A preliminary study of two methods of weaning foals. Appl. Anim. Behav. Sci..

